# WRAP-based nanoparticles for siRNA delivery: a SAR study and a comparison with lipid-based transfection reagents

**DOI:** 10.1186/s12951-021-00972-8

**Published:** 2021-08-11

**Authors:** Karidia Konate, Emilie Josse, Milana Tasic, Karima Redjatti, Gudrun Aldrian, Sébastien Deshayes, Prisca Boisguérin, Eric Vivès

**Affiliations:** 1 PhyMedExp - Université de Montpellier, INSERM U1046, CNRS UMR 9214, CHU Arnaud de Villeneuve, 371 av. doyen Giraud, 34295 Montpellier Cedex 5, France; 2 Sys2Diag, UMR 9005-CNRS/ALCEDIAG, 1682 Rue de la Valsière, 34184 Montpellier CEDEX 4, France

**Keywords:** Cell-penetrating peptides, Nanoparticle, siRNA delivery, Structure–activity relationship

## Abstract

Recently, we designed novel amphipathic cell-penetrating peptides, called WRAP, able to transfer efficiently siRNA molecules into cells. In order to gain more information about the relationship between amino acid composition, nanoparticle formation and cellular internalization of these peptides composed of only three amino acids (leucine, arginine and tryptophan), we performed a structure–activity relationship (SAR) study. First, we compared our WRAP1 and WRAP5 peptides with the C6M1 peptide also composed of the same three amino acids and showing similar behaviors in siRNA transfection. Afterwards, to further define the main determinants in the WRAP activity, we synthesized 13 new WRAP analogues harboring different modifications like the number and location of leucine and arginine residues, the relative location of tryptophan residues, as well as the role of the α-helix formation upon proline insertions within the native WRAP sequence. After having compared the ability of these peptides to form peptide-based nanoparticles (PBNs) using different biophysical methods and to induce a targeted gene silencing in cells, we established the main sequential requirements of the amino acid composition of the WRAP peptide. In addition, upon measuring the WRAP-based siRNA transfection ability into cells compared to several non-peptide transfection agents available on the markets, we confirmed that WRAP peptides induced an equivalent level of targeted gene silencing but in most of the cases with lower cell toxicity as clearly shown in clonogenic assays.

**Supplementary Information:**

The online version contains supplementary material available at 10.1186/s12951-021-00972-8.

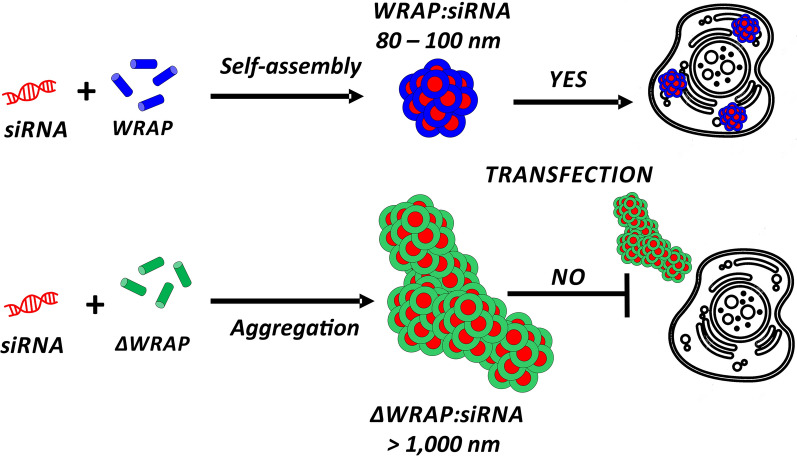

## Introduction

Transfection of siRNAs is nowadays widely employed to specifically knock-down the cellular expression of any targeted protein. However, siRNAs are unable to translocate within the cells without the use of transfection reagents. After the development of various compounds such as cationic lipids, polymeric molecules or inorganic nanoparticles to promote their cell delivery, peptides called cell-penetrating peptides (CPPs) have been also designed and used alternatively. During the last years, several parameters have been investigated to understand how CPPs could be more efficient in transferring into cells different types of nucleic acids (plasmids, siRNAs, etc.) [1, 2]. Therefore, different substitutions, deletions and modifications within their primary sequence have been performed and the translocating activities of the corresponding peptides have been compared to the parental CPPs. These investigations included the number of arginine residues [3], the presence and the location of tryptophan residues within the sequence [4, 5], the integration of apolar moieties [6, 7] either as non-natural amino-acids [8], as fatty acids [9, 10] or as hydrophobic groups directly grafted onto the CPP [11–14]. Structural studies have been also conducted to define whether the CPP secondary structure could influence the translocating process [15]. By biophysical methods, we confirmed with one of these CPPs, CADY-K, that α-helix structuration was an important prerequisite for stable peptide-based nanoparticles (PBNs) formation with siRNAs [16]. The previously shown structural polymorphism of amphipathic peptides (helicoidal structure formation in a specific environment) [17] was also demonstrated using a fully *retro-inverso* peptide [18]. Another group suggested that amphipathic helical peptides composed of hydrophobic and cationic amino acids exposed on different sides of the helix could be typical CPPs for driving the uptake of siRNAs [19]. We recently showed that peptides composed only of arginine, leucine and tryptophan residues presented a random/helical structure in water with the ability to attain a helical conformation in the presence of anionic components (nucleic acids or membrane-mimicking compounds) [20]. This observation has been also reported by Chen’s group who designed a peptide which showed a random/helical structure in water with the ability to attain a helical conformation upon interactions with anionic components or membrane-mimicking environments [21]. Therefore, all these observations could focus towards a crucial amphipathic structuration of CPPs for inducing the uptake of at least this class of short nucleic acid molecules, namely siRNAs.

It is also widely admitted that the inability of nucleic acids to enter cells also relied on their anionic nature upon interferences with the negatively charged glycoproteins of the cell membrane and with the anionic components of the lipid bilayer itself. Consequently, there was also a clear evidence that cationic amino acids within CPP sequences were important for the formation of PBNs, likely through ionic interactions with negatively charged molecules such as nucleic acids. Along this line, we and others notified a stoichiometry between the CPPs and the nucleic acids to ensure its full complexation, leading to a positive zeta potential of the PBNs [20–22]. Indeed, positive zeta potential of PBNs should reflect the complete neutralization of anionic charges of the nucleic acids, but thanks to the overall excess of cationic charges, should also favor interactions with anionic cell surface components, and ultimately, their potential cellular uptake.

To neutralize these anionic charges, we integrated logically cationic amino acids in our last family of tryptophan- (W) and arginine-(R) rich Amphipathic Peptides (WRAP) peptides [20]. There are three natural cationic amino acids available (arginine, lysine and histidine), each in depth investigated for their relative influence on the translocating activity of CPP. For instance, it has been demonstrated that histidine residues could play a role in the swelling and disruption of endosomes, thus allowing nucleic acids to enter the cytoplasm following endocytosis. However, in a complete study about the mechanism of entry of our WRAP series using endocytosis markers, endocytosis inhibiting conditions and transmission electron microscopy (TEM) [23], we showed that the internalization pathway of our siRNAs-WRAP PBNs was mainly a direct membrane translocation, thus mostly avoiding the endosomal pathway. Therefore, no benefit should be expected from the integration of histidine residues within our WRAP peptides to develop efficient siRNAs transporters. The literature also abounds in demonstrations of the key role of arginine residues in the translocating properties of CPPs. This has been highlighted following deletion of arginine residues within the Tat peptide, one of the first CPPs to be discovered [24]. Few years later, efficient CPPs made of 6–10 arginine residues only have been shown to be internalized in cells [25], thus encouraging the integration of arginine residues in CPP primary sequences. It has been also quantified that the substitution of arginine residues by lysine, ornithine or histidine residues within CPP sequences reduced dramatically their ability to enter cells [26]. Consequently, we integrated arginine residues only in these CPPs.

Another amino acid which appeared to be important for promoting the translocation properties of CPPs was the tryptophan residue [27]. When a tryptophan residue was replaced by a tyrosine, the loss of cellular transfection of siRNAs was recorded, surprisingly despite the formation of much smaller nanoparticles [28]. The importance of tryptophan residues has been also confirmed with a Penetratin analogue only composed of arginine and tryptophan [29]. Interestingly, a corresponding 9-mer peptide made only with arginine and leucine was no more taken up by cells, thus confirming the peculiar role of tryptophan residues. We also showed that the reduction of the number of tryptophan residues in WRAP sequences impaired directly PBNs formation (with aggregates over 1,000 nm of diameter), and consequently, their cell translocation activity [20]. In this latter work, we also determined leucine residues to be more efficient than glycine or alanine residues for promoting the formation of effective siRNAs-WRAP PBNs [20]. Therefore, we considered the WRAP peptide series to be interesting peptides to further define the requirement towards efficient CPPs for inducing siRNA delivery in a wide panel of different cell lines [20].

The primary sequence and amino composition (with only leucine, arginine and tryptophan residues) of this series of WRAP peptides we first designed [20] was close to those of the C6M1 peptide and its leucine analogue published by Chen’s group [21]. Therefore, there was the need to perform a structure–activity relationship (SAR) study, from the initial formation of the PBNs until the ability of the different PBNs to induce a biological response of the siRNAs. The importance of different features (location and number of these amino acids within WRAP peptide sequences and structural content) to induce the higher siRNAs transfection response had to be further investigated.

Finally, since the main use of such transfecting peptides is motivated by the cellular transport of nucleic acids, either plasmids or siRNAs, we wished to evaluate the efficacy and the toxicity of our WRAP peptides series compared to the main siRNA transfecting agents available on the market.

## Results

### Comparison of WRAP and C6M1: two CPPs with a close amino acid composition

**Fig. 1:**
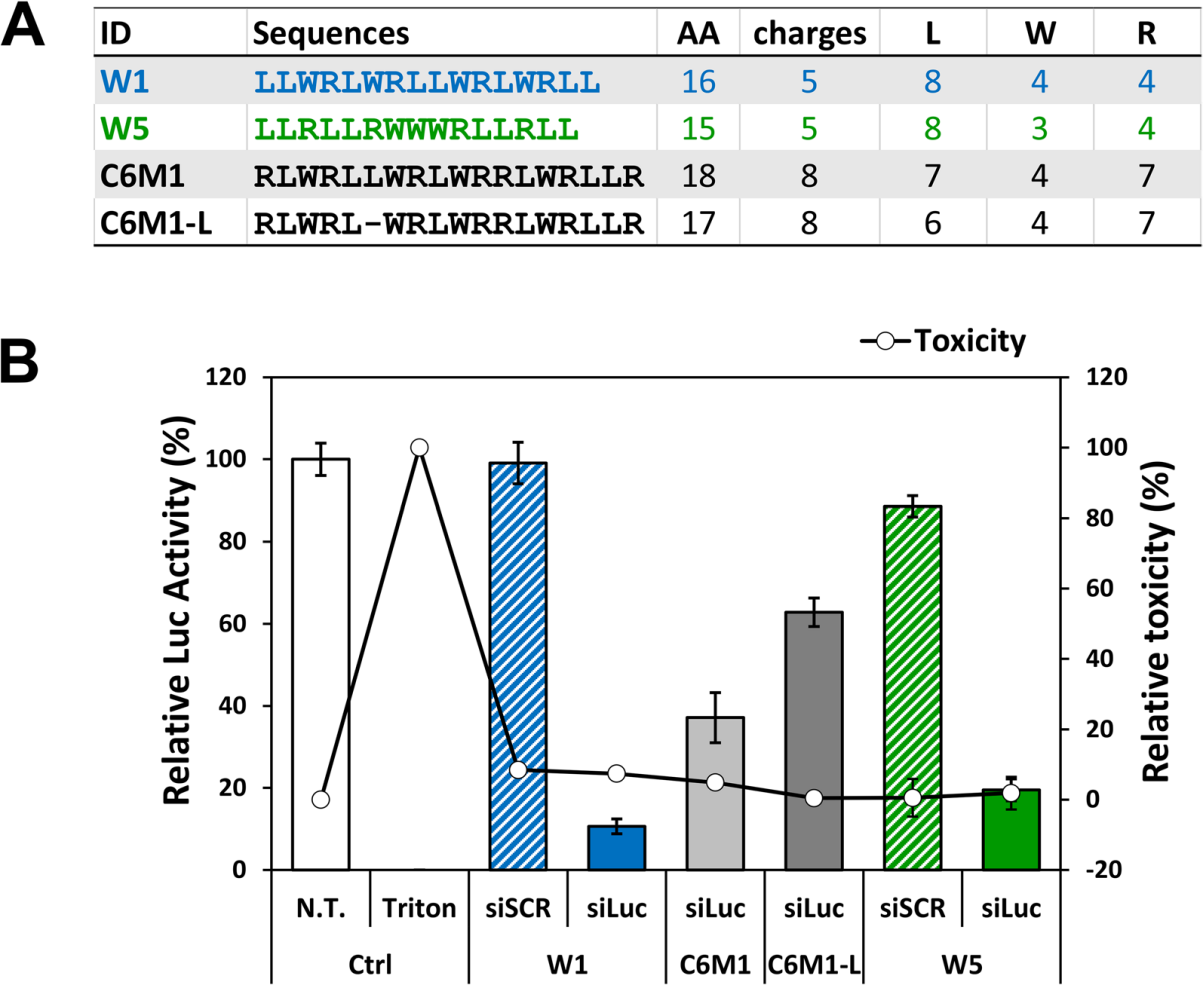
Comparison of WRAP and C6M1 cell-penetrating peptides. **A** Table summarizing the WRAP and C6M1 peptide sequences and their characteristics. **B** Graphical representation of the relative Luc activity (%) and relative cytotoxicity (LDH quantification, %) after transfection with CPP:siRNA complexes on U87 cells. Conditions: CPP:siRNA (R = 20) using a siRNA concentration of 10 nM and a peptide concentration of 200 nM. Abbreviations: siLuc = firefly luciferase siRNA, siSCR = scrambled version of the siLuc, N.T. = non-treated cells, Ctrl = Controls. Data represent mean ± SD, with n = 2 independent experiments in triplicate

To get more insight on the role of amino acid composition during the transfection of siRNAs, we compared WRAP1 (W1) and WRAP5 (W5) with the C6M1 peptide developed by Chen’s group [21, 30]. Additionally, we implemented the C6M1-L peptide resulting from a synthesis deletion. These four peptides have nearly the same amount of tryptophan residues (three or four depending on the peptide) but differ more importantly in the amount of arginine (four for W5 and W1 and seven for C6M1) or leucine residues (six to eight depending on the peptide). All together these resulted in longer peptides in combination with a higher number of charges for C6M1/C6M1-L compared to the WRAP series (Fig. 1A).

To evaluate the biological activity of WRAP-PBNs in comparison with C6M1/C6M1-L-PBNs, we used human glioblastoma U87 cell line stably transfected for constitutive expression of FLuc/NLuc reporter genes to observe the potent luciferase silencing. Furthermore, even if the WRAP-PBNs were able to induce protein silencing in the presence of serum [20], we performed all PBN incubations in serum-free medium (1.5 h) followed by the addition of serum-containing medium (36 h).

In Fig. 1B, we observed for both peptides C6M1 and C6M1-L a lower silencing activity (60% and 35%) compared to both WRAP peptides (80%–90%) encapsulating in each case the same amount of siRNA (R = 20, [Peptide] = 200 nM, [siRNA] = 10 nM). Considering the close similarity in amino acids content between the two families of peptides and differences in the primary sequence, this result suggested that the amount of leucine and arginine residues were important for the silencing activity of the resulting PBNs. This could be the consequence of the higher number of arginine in the C6M1 series (7) compared to the WRAP series (4). Therefore, we could formulate objectively the hypothesis that a higher content of arginine was unfavorable for a strong biological response. Indeed, in our biological assay, we measured a global effect, ranging from the PBNs formation till the siRNA effects at the RISC system. In between, the PBNs have to internalize the cells and siRNAs have to be properly released from the PBNs. A lower release for the C6M1 PBNs could explain a weaker availability of siRNA molecules and consequently a lower biological response because of the higher content of ionic interactions. In line with this, we clearly observed the direct influence of arginine residues in the release rate of siRNAs from PBNs made of WRAP peptides with different amount of arginine residues (see below).

Another noticeable difference was the lower number in leucine residue for the C6M1 peptide series (6 or 7) compared to the WRAP series (8). A lower number of leucine residues seemed to correlate with a lower biological response. Along this line, we observed that the deletion of one leucine in the C6M1-L peptide compared to the native C6M1 peptide seemed to further reduce the silencing property of the PBNs (Fig. 1B). Therefore, we investigated whether the N- and C-terminal and internal leucine doublets in our WRAP peptide series were indispensable all along the different steps from the nanoparticle formation towards the recorded gene silencing activity.

### Dissecting the role of the leucine doublets within the WRAP sequence

To confirm this hypothesis, we synthesized analogues deleted of one leucine located at both N- and C-terminal regions of the WRAP peptides (W1-2L and W5-2L, respectively). Additionally, we synthesized the W5-2Lm peptide to analyze the role of the intra-sequential leucine doublets and the W5-4L peptide with all possible leucine doublet deletions (Table 1).

**Table 1:**
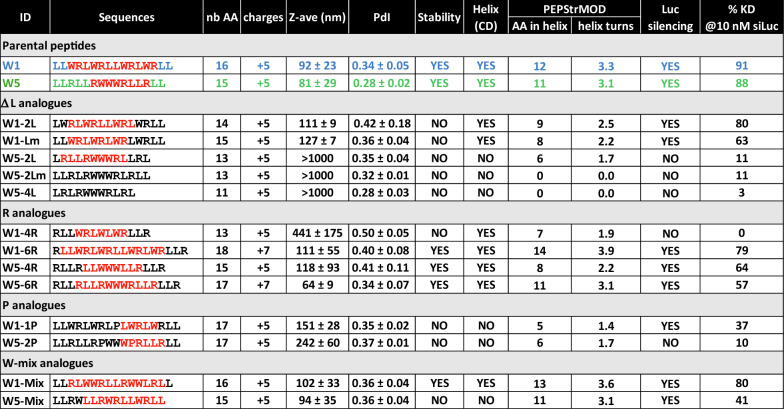
Characterization of the WRAP peptides and their analogues used in this study

Represented in the table are the primary sequences (red = amino acids implicated in the helix according to PEPstrMOD prediction), the number of amino acids (AA), the number of charges, the mean size [Z-average, nm] and the polydispersity index (PDI), the stability of the PBNs after a 4 day-period at 4°C, the helix signal in CD measurements, the number of amino acids implemented in the helix (AA in helix), and the calculated number of helix turn (number of amino acids in the helix divided by 3.6 as predicted by PEPStrMOD calculation), the luciferase silencing activity and the level of silencing in percentage at the dose of 10 nM siRNA

**Fig. 2:**
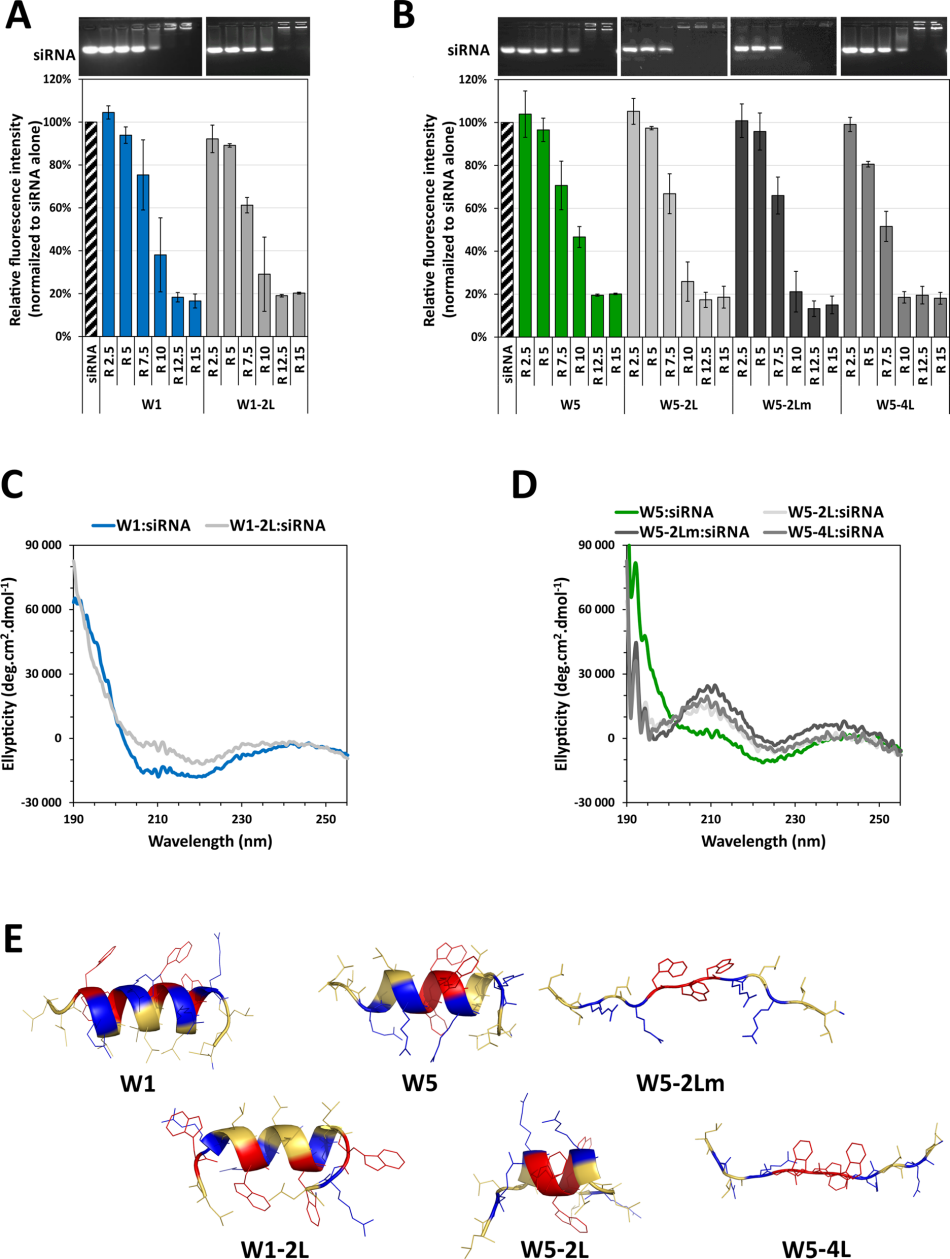
Evaluating the influence of leucine doublets within the WRAP sequences. **A** and **B** Evaluation of the capacity of the WRAP peptides and WRAP analogues to form complexes in the presence of siRNAs (3.5 µM). Preformed CPP:siRNA complexes at different molar ratio (R) were analyzed by electrophoresis on agarose gel. Data represent mean ± SD, with n = 3 independent experiments. **C** and **D** Representation of the CD spectra of the CPP:siRNA complexes (R = 20, [CPP] = 10 µM, [siRNA] = 0.5 µM). **E** Ribbon presentation of the predicted 3D structure of the WRAP peptides and WRAP-analogues by PEPstrMOD in a hydrophilic environment

Both parental peptides, W1 and W5, were compared to their respective Leu-analogues by agarose shift assay in order to verify first their ability to complex siRNAs (Fig. 2A and B). Without the peptide addition, siRNAs [3.5 µM] migrated into the agarose gel (= 100% signal). But when complexed with an increased molar ratio of W1 or W5 peptides [8.75 µM, 17.5 µM, 26.25 µM, 35 µM, 43.75 µM and 52.5 µM, for molar ratio of R2.5, R5, R7.5, R10, R12.5 and R15, respectively], siRNA migration was prevented in a molar ratio-dependent manner as previously reported [20] (Fig. 2A and B). W1-2L, W5-2L, W5-2Lm and W5-4L clearly complexed siRNAs similarly to the parental peptides, with a complex formation starting at a molar ratio of 10 (R = 10 for > 50% of complexed siRNAs). However, because we [20] and others [30] showed that higher molar ratios were more stable (Additional file 1: Figure S1) and more suitable for an *in cellulo* application, we decided to use the molar ratio R = 20 for PBN formulations in all experiments. Additionally, biological assays were performed using the same PBN solutions studied by CD and DLS experiments. This allowed us to directly correlate the PBN formation with the biological activity, thus avoiding potential artifacts due to the preparation of different formulations.

As reported previously [20], WRAP peptide solutions were nearly unstructured on their own (W1 = random coiled and W5 = turn conformation). They adopted an α-helical conformation once incubated with siRNAs. Circular dichroism (CD) measurements were performed for all peptide analogues, both alone [10 µM] and in the presence of siRNAs [0.5 µM] to verify whether leucine doublet deletions would influence any conformational switch (Fig. 2C and D and Additional file 1: Figures S2 and S3). W1-2L revealed a conformational switch showing the tendency of a helical structure formation (increased maxima at 195 nm and induced minima at 202 nm or 227 nm) (Fig. 2C). In contrast, W5-2L, W5-2Lm and W5-4L remained mainly unstructured with the presence of the two minima (203 and 227 nm) suggesting a turn conformation as for W5 (Additional file 1: Figure S3A–D). Upon incubation with siRNAs, the tryptophan cluster contribution, corresponding to the minima around 227 nm, was maintained for all mutants but the lack of leucine gave rise to a small maximum at 210 nm (Fig. 2D). To better understand these differences, we performed in silico 3D structure prediction of WRAP and analogue peptides alone (Fig. 2E). After computation, the peptide models generated by PEPstrMOD [31, 32] in a hydrophilic environment revealed that W1, W5, W1-2L were able to adopt α-helical structure. W5-2Lm and W5-4L had no helical structure, confirming the CD measurements. It is noteworthy to mention that the leucine-deleted peptides W5-2L was predicted to adopt a short helix, which is in contradiction to the CD evaluation. However, in case of discrepancies between the measured and the expected results about the helical content within the peptides, it is worth noticing that we were more confident with the results provided experimentally by CD measurements than with those predicted by a theorical modelling system.

Afterwards, colloidal features of WRAP:siRNA complexes (R = 20) were characterized by Dynamic Light Scattering (DLS) in terms of nanoparticle size and polydispersity of size distribution (same solution than those used for the CD measurement). Intensity measurements (%) revealed that W1 and W5 formed PBNs with diameters of 80–100 nm with a polydispersity index (PdI) around 0.3 (Table 1) as reported previously [20]. A comparable result could be observed for W1-2L, showing a mean size of 111 nm with a PdI of 0.42. In contrast, W5-2L, W5-2Lm and W5-4L showed mean sizes higher than 1000 nm indicating that these three peptides were not able to form PBNs in the presence of siRNAs.

Based on these results, we could conclude that leucine doublets deletion impaired directly the nanoparticle formation in the presence of siRNAs. Even if W5-2L was susceptible to form a short α-helix as revealed by molecular modelling (Fig. 2E), whether the peptide was analyzed alone or complexed with the siRNA, CD measurements did not indicate any helical structuration to ensure the formation of stable PBNs (Additional file 1: Figure S3B). This was confirmed by the DLS measurements showing the formation of aggregates (size in the µm range) and not of defined nanoparticles. In contrast, the deletion of the N- and C-terminal leucine doublets in the W1 peptide did not prevent the formation of an α-helical structure and resulted in W1-2L:siRNA nanoparticles with a mean size of 111 nm.

### Evaluating the role of arginine residues within the WRAP sequence

Since the C6M1 peptide harbored arginine residues at both extremities, we wanted to evaluate the effect of arginine residue addition at both N- and C-terminal ends of the WRAP sequences by synthesizing analogues with one additional arginine residues at both ends of the peptide (Arg = 6 for W1-6R and W5-6R). However, because the comparison between C6M1 and WRAP peptides suggested the importance to keep an identical amount of arginine residues in order to maintain a good biological effect (Fig. 1B), we designed peptides with two additional arginine residues at both extremities, but with two arginine residues less within the sequence to keep the same charge number as the parental peptides (Arg = 4 for W1, W5, W1-4R and W5-4R) (Table 1).

**Fig. 3:**
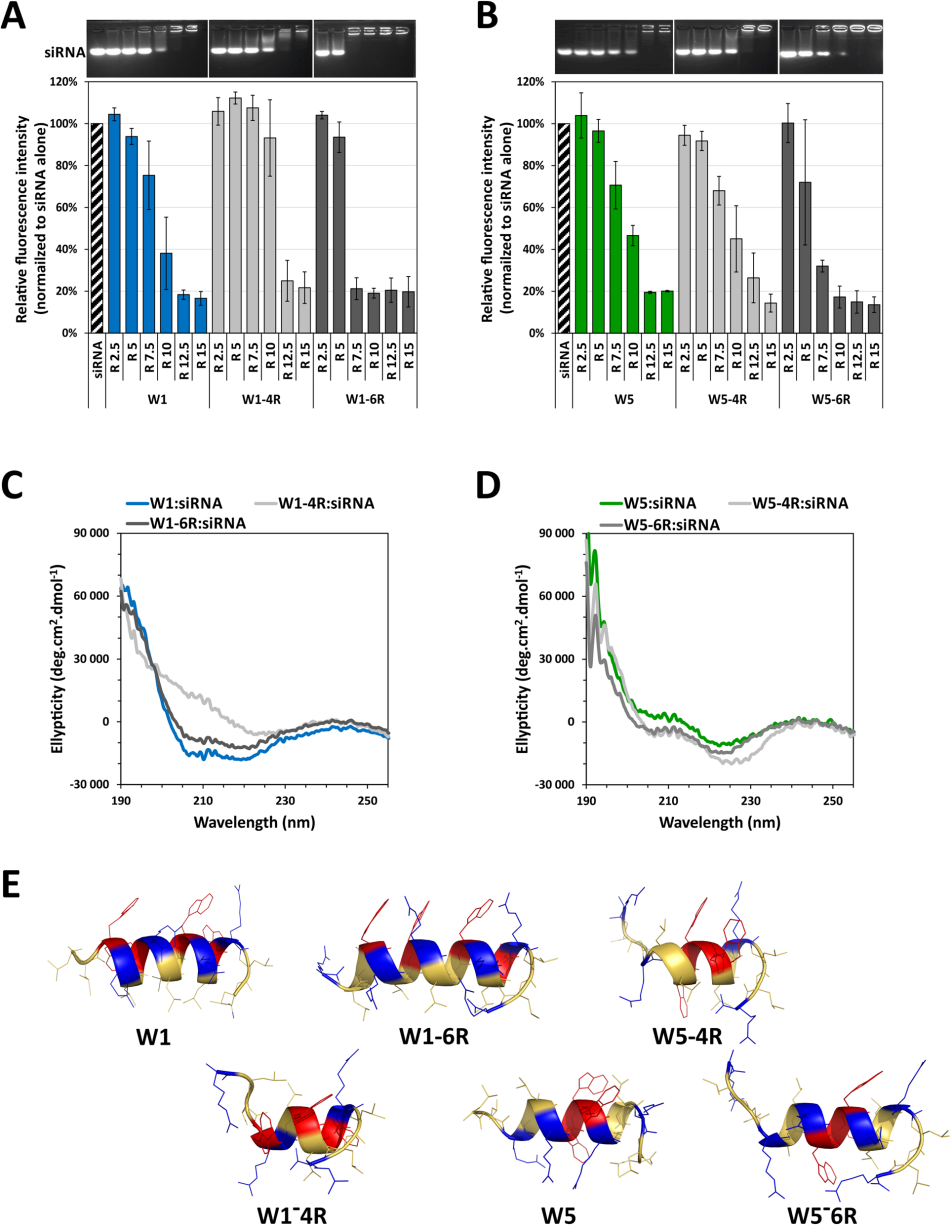
Evaluating the influence of the number of arginine residues within the WRAP sequences. **A** and **B** Evaluation of the capacity of WRAP peptides and WRAP analogues to form complexes in the presence of siRNAs (3.5 µM). Preformed WRAP:siRNAs complexes at different molar ratio (R) were analyzed by electrophoresis on agarose gel. Data represent mean ± SD, with n = 3 independent experiments. **C** and **D** Representation of the CD spectra of the WRAP:siRNA complexes (R = 20, [CPP] = 10 µM, [siRNA] = 0.5 µM). **E** Ribbon presentation of the predicted 3D structure of the WRAP peptides and WRAP-analogues by PEPstrMOD in a hydrophilic environment

As performed above for the ∆Leu-analogues, we first evaluated by agarose gel shift assay the ability of these Arg-analogues to form PBNs in the presence of siRNAs depending on the used molar ratio required for an optimal siRNA complexation (same conditions as above) (Fig. 3A). Increasing the number of arginine residues clearly improved complex formation compared to the parental peptides (molar ratio R = 7.5 for W1-6R and W5-6R versus R = 10 for W1 and W5, respectively). This was likely related with the higher number of positive charges in peptides containing 6 arginine residues. In contrast, the simultaneous N-and C-terminal arginine addition together with the internal arginine deletion seemed to slightly impact negatively nanoparticles formation as revealed by the higher molar ratio required to fully complex the siRNA (R = 12.5 for W1-4R and W5-4R). This could be related with the lower biological effect observed for the C6M1 peptides compared to the WRAP peptides (Fig. 1B) and to the hypothesis of a weaker release ability for peptides containing a higher number of arginine residues. On the other hand, the CD spectra showed in both cases that W1-6R and W5-6R adopted in the presence of siRNAs a helical conformation comparable to those recorded with the parental peptides (Fig. 3C and D). For the W1-4R siRNA-loaded complexes, we observed some slight changes in the helical structure while the lack of arginine inner residues of W5-4R did not give any conformational change compared to W5 in the presence of siRNA. Structure predictions by PEPstrMOD [31, 32] in a hydrophilic environment revealed that the potential helix was shorter for W1-4R and W5-4R compared to the other peptides (Fig. 3E). These behaviors could indeed impair the capacity of these peptides to form stable PBNs with the siRNA.

As expected from the results above, all Arg-analogues complexed with siRNAs formed PBNs with mean sizes in the 100 nm range as measured by DLS (Table 1). However, we observed for W1-4R slightly bigger (441 ± 175 nm) and more dispersed PBNs (0.50 ± 0.05).

In conclusion, adding two arginine residues to the N- and C-terminal end of WRAP sequences seems to induce PBN formation at lower molar ratio without perturbing importantly the size of the formed PBNs. Displacing the two internal arginine residues at the N- and C-terminal end of WRAP sequences (W1-4R and W5-4R—same number of arginine residues as the parental peptides) resulted in minimal conformational changes and PBN formation, with the exception of W1-4R which formed four times bigger PBNs.

### Comparison of the luciferase silencing activity of the WRAP analogues

**Fig. 4:**
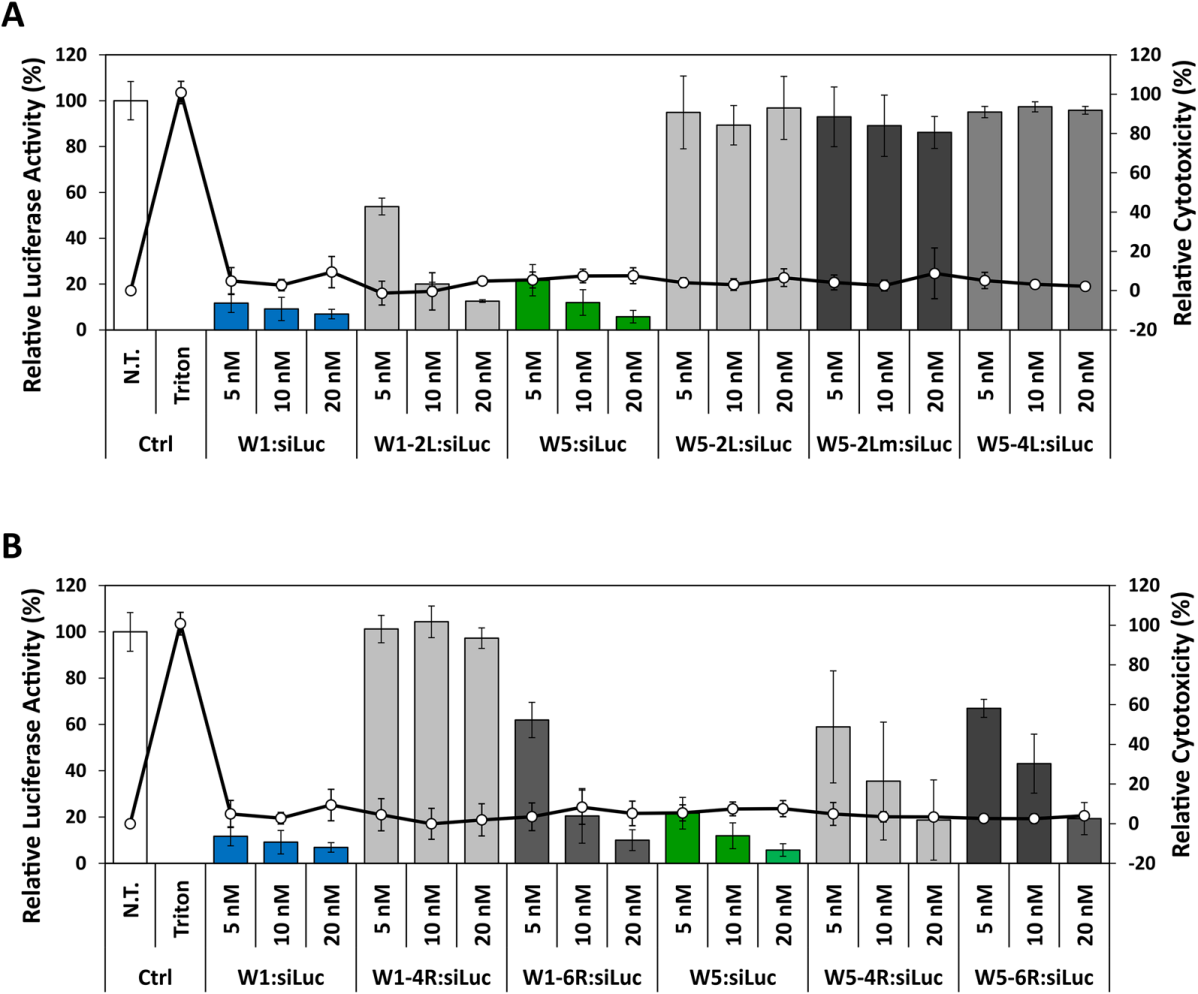
Comparison of luciferase silencing of WRAP-based PBNs. **A** ∆Leu-analogues and (**B**) Arg-analogues complexed with siRNA as PBNs were evaluated for their luciferase silencing activity in comparison to the parental W1 and W5 PBNs. Graphical representation highlighted the relative Luc activity (%) and relative cytotoxicity (LDH quantification, %) after transfection with WRAP:siLuc complexes on U87 cells. Conditions: WRAP:siRNA (R = 20) at the indicated siRNA concentrations. Abbreviations: siLuc = firefly luciferase siRNA, N.T. = non-treated cells, Ctrl = Controls. Data represent mean ± SD, with n = 2 independent experiments in triplicate

Cellular activity of the different WRAP PBNs was evaluated on a luciferase positive human glioblastoma U87 cell line. To this aim, PBNs solutions used for CD and DLS measurements were diluted to siRNA concentrations of 5 nM, 10 nM and 20 nM (R20, [peptide] = 100 nM, 200 nM and 400 nM, respectively) and directly applied on cells to perform the luciferase assay. W1:siRNA and W5:siRNA gave impressive silencing activities for the three siRNA concentrations (Fig. 4A, B and Table 1). We noticed that these inhibitions were higher than inhibition levels previously reported [20]. For instance, we obtained before around 60% luciferase activity remaining with 5 nM siRNA concentration compared here to 10%. Interestingly, we found out that this disparity resulted from the differently formulated PBNs. The knock-down efficiency of siRNA-loaded WRAP PBNs appeared more efficient when PBNs were first formulated at high concentration and then afterwards diluted, compared to those formulated directly at the desired concentrations (Additional file 1: Figure S4). At this moment, we do not have any rational explanation about this phenomenon and we are performing studies to understand this factual result.

However, because all PBNs were formulated and diluted in the same way, the silencing efficiency could be directly compared. W1-2L:siRNA showed a reasonable luciferase knock-down activity even if the effect was not so pronounced compared to W1:siRNA. In contrast, none of the three W5 ∆L-analogues induced any luciferase silencing (Fig. 4A).

All W1-6R, W5-4R and W5-6R siRNA-loaded PBNs showed a dose-dependent luciferase silencing, but slightly less important compared to the parental WRAP PBNs (Fig. 4B). In contrast, with a bigger PBN size compared to parent peptide, W1-4R:siRNA had no activity at all used siRNA concentrations.

By looking in details all measured characteristics, we found out that the length of the peptide helix formed in the presence of siRNAs could be a favorable indicator for the PBN silencing activity (Table 1). Knowing that a typical α-helix contains 3.6 amino acids per helical turn [33], we simply calculated the amount of helix turns in the helix depending on the number of amino acids implicated in the helix formation (highlighted in red in the primary sequence in Table 1). Interestingly these numbers directly correlated with the level of the luciferase silencing. No luciferase silencing activities were measured for peptides forming less than two helical turns (W1-4R and W5-2L). However, if more than two helical turns were present in the peptide, we observed an important luciferase silencing activity suggesting that this minimal helix length could be crucial for stable PBN formation and efficient cellular translocation.

However, the overall length of the helical structure in the PBNs did not fully highlight a clear correlation with an optimal silencing efficacy. For example, W1-6R:siRNA with nearly four helical turns showed less silencing activity than the parental W1 peptide (79% vs 91% of knock-down, respectively). A similar effect was observed for W5-6R:siRNA, with a number of helical turns (3.1) comparable to those of the parental peptide W5. In this latter case, the silencing activity dropped down from 88% for W5 to 57% for W5-6R, respectively. To confirm that arginine residue addition had a negative impact on PBN activity, we performed heparin competition experiments to check the stability of the different PBNs. Heparin are sulfated polysaccharide molecules, highly present on the extracellular matrix of cells, which could be able to interact with positive charges of peptides contributing thereby to the siRNA release. The heparin sensitivity of PBNs provoking their instability (Additional file 1: Figure S5) could explain the lack of silencing activity. Compared to the parental peptides W1 and W5, only W5-2L and W1-4R showed a higher level of destabilization in good correlation with the lack of silencing activity (Table 1). Whether this lower transfection efficacy was the result of a lower release of the siRNA at the cell membrane level or within the cell remains to be fully assessed.

For the analyzed WRAP PBNs and a selection of their analogues (W1-2L, W1-4R, W5-2L and W5-4R), we observed by confocal microscopy that the luciferase silencing properties correlated strongly with the peptide-based internalization of the siRNA-Alexa488 (Additional file 1: Figure S6). In details, no fluorescence was observed for the siRNA alone as well as for W1-4R:siRNA and W5-2L:siRNA. Fluorescence intensity was higher for the W1:siRNA and the W5:siRNA compared to W1-2L:siRNA and W5-4R:siRNA in analogy to the luciferase silencing.

Additionally, we evaluated the relative cytotoxicity of the analyzed WRAP PBNs in parallel of the luciferase assay, showing that none of them induced any effect on the U87 cells (> 20% of cytotoxicity) (Fig. 4A, B). Finally, we analyzed cell viability and cytotoxicity of the WRAP peptides alone (Additional file 1: Figure S7) in a concentration range of 0.125 µM to 2 µM. These concentrations corresponded to concentrations used to formulate PBNs made with siRNAs at the concentration of 6 nM to 100 nM (molar ratio of R20). For nearly all peptides, we observed no effect on cell viability or cytotoxicity using 0.125 and 0.5 µM, some impact depending on the used peptide at 1 µM and high effects at 2 µM concentration. However, previously reported membrane leakage made with PBNs showed lower toxicity than those observed with the peptide alone [20]. Therefore, we estimate that effects on cell viability will be identically lower once the peptides were complexed with the siRNA.

### Evaluating the role of the helix formation in the WRAP sequences

To evaluate the importance of the helical structure, and more particularly of its length within the WRAP sequences on PBN formation, we synthesized a new peptide set by integrating one or two proline residues in the middle of their sequences. Indeed, proline residues are unable to form hydrogen bonds within an alpha-helix structure because of the lack of hydrogen on their amide nitrogen. Therefore, proline residues are well-known perturbators of helical structures [33].

**Fig. 5:**
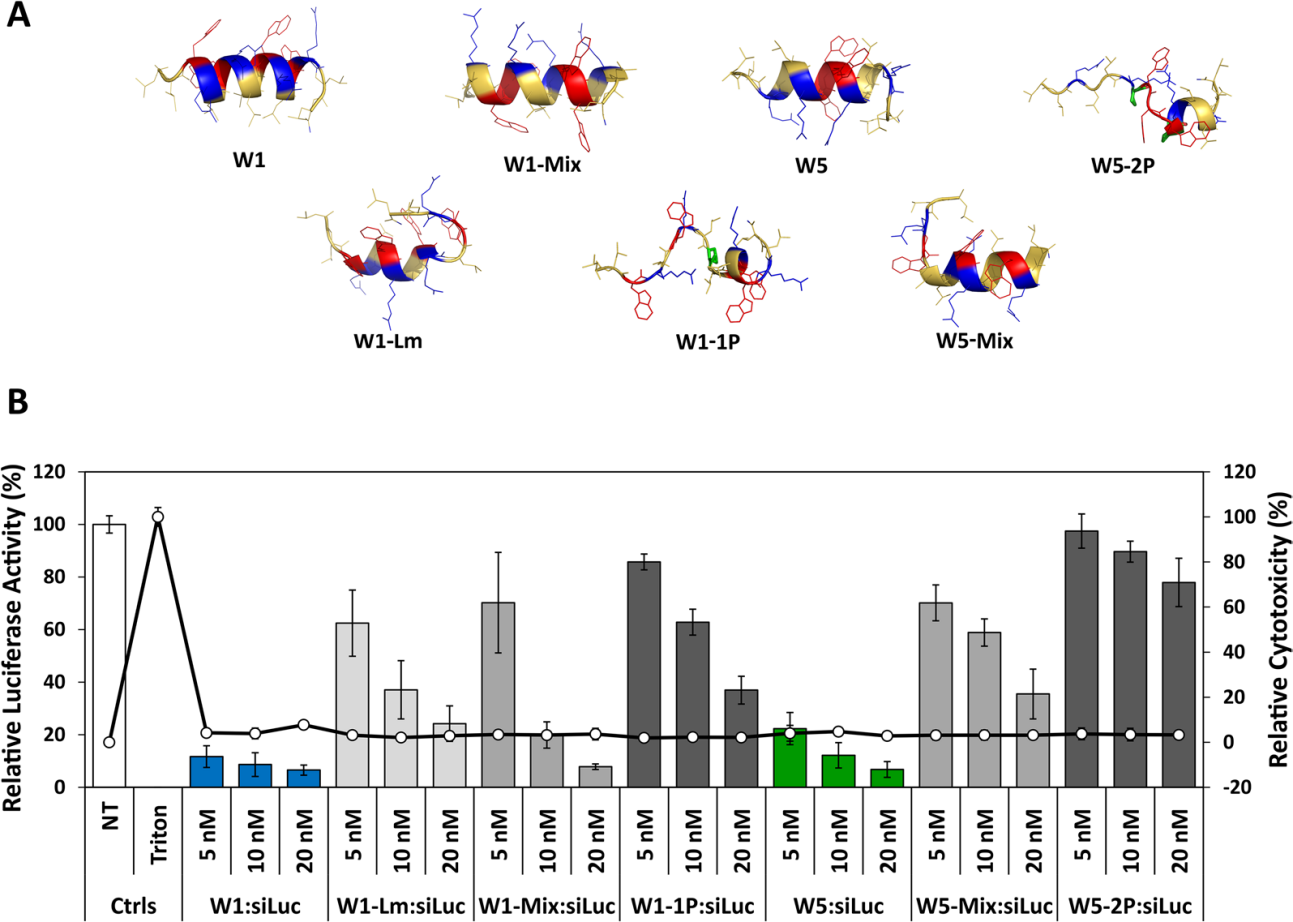
Comparison of structure and luciferase silencing of WRAP-based PBNs and their analogues. **A** Ribbon presentation of the predicted 3D structure of the WRAP peptides and WRAP analogues by PEPstrMOD in a hydrophilic environment. **B** siRNA-loaded WRAP analogues were evaluated for their luciferase silencing activity in comparison to the parental WRAP PBNs. Graphical representation highlighted the relative Luc activity (%) and relative cytotoxicity (LDH quantification, %) after transfection with WRAP:siLuc complexes on U87 cells. Conditions: WRAP:siRNA (R = 20) at the indicated siRNA concentrations. Abbreviations: siLuc = firefly luciferase siRNA, N.T. = non-treated cells, Ctrl = Controls. Data represent mean ± SD, with n = 2 independent experiments in triplicate

As expected, both proline-containing peptides (W1-1P and W5-2P) showed nearly no structural features, whether the analysis was performed using molecular 3D structure prediction (PEPstrMOD) or using circular dichroism analyses (Figs. 5A, Additional file 1: Fig. S1 and S2). In details, CD analyses did not show any major structural changes whether analyses were performed on the peptides alone ([peptide] = 10 µM) or associated with siRNAs ([peptide] = 10 µM, [siRNA] = 0.5 µM) (Additional file 1: Figures S1F and S2G). Once mixed with siRNAs, both peptides W1-1P and W5-2P still formed PBNs, but with diameters of 151 ± 28 nm and 242 ± 60 nm, respectively, slightly bigger than those of the PBNs formed with the corresponding parental peptides (80–100 nm range, see Table 1). When evaluating their luciferase knock-down activity, W1-1P and W5-2P were shown to be 6 to 9 times less efficient, respectively, compared to the parental peptides (Fig. 5B). Whether the slight increase in PBN size was responsible for this significant reduction luciferase silencing induced by the siRNA-loaded PBNs remains difficult to be clearly distinguished.

Because the central “RLLRSL” motif of the CADY peptide sequence initiated the helical structure in all hydrophobic media [34], we evaluated whether the central leucin doublet could also have an impact on the W1 helix formation by synthetizing a W1 analogue having only one leucin residue in the middle of the sequence (W1-Lm). This ∆-L peptide analogue showing a low signal of helical structure (minimum at 208 and 222 nm with molar ellipticity values under −10,000 deg.cm^−2^.dmol^−1^) as shown by CD measurements (Additional file 1: Figure S2C) was an accordance with the PEPstrMOD prediction which revealed a 2.2 turns helix (Fig. 5A). Furthermore, the formed W1-Lm:siRNA complexes with a mean size of 127 ± 7 nm could induce a luciferase activity silencing of 63% ([siRNA] = 10 nM, [W1-Lm] = 200 nM) Fig. 5B, Table 1) revealing that the deletion of the internal leucin doublet could impact the size (higher) and the activity (lower) of the PBNs.

Finally, we also synthesized W1-mix and W5-mix analogues in which the tryptophan residues were displaced regarding their location in the parental peptides in order to evaluate their influence on the helix formation. Even, if the helicity of the peptides in the presence of siRNA was fully maintained (Additional file 1: Figures S2G and S3H), we observed a slightly higher mean size diameter for these PBNs compared to the parent peptides. Concerning the biological effects of these W-mixed peptides, we were surprised to observe differences: W1-mix:siRNA revealed a luciferase knock-down activity of 80% compared to W5-mix:siRNA having only 41% ([Peptide] = 200 nM, [siRNA] = 10 nM) (Fig. 5B, Table 1). These results implicated that 4 tryptophan residues could be dispatched along the peptide sequence but that 3 tryptophan residues should be preferably grouped to obtain a comparable luciferase silencing property.

As previously shown for the ∆Leu and Arg-analogues, the silencing efficiency of the here analysed WRAP analogues correlated with their internalization properties (Additional file 1: Figure S6). In details, fluorescence-labelled siRNA transfected with W1-mix and W5-mix revealed a lower cellular internalization than the parental peptides, and a very lower internalisation for W1-1P and W5-2P.

### WRAP nanoparticles compared to lipid-based reagents for siRNA transfection

Finally, WRAP peptides were compared with other commercially reagents available for siRNA transfection such as RNAiMAX (Life Technologies), INTERFERin (PolyPlus), DharmaFect (Dharmacon) and HiPerFect (Qiagen). They were selected from their publication score in PubMed and they were evaluated for their siRNA transfection capacity (luciferase assay) and their potential cytotoxicity (clonogenic assay and trypan blue).

However, because protocols of each transfection reagent recommended by the manufacturers were different in terms of cell density, amount of transfection reagent, volume of the transfection mixture, incubation volume and used media (see Table 1-SI), we decided to apply the recommended amount of each transfection reagent, but in combination with the WRAP-PBN incubation protocol (initial incubation for 1.5 h with serum free medium followed by the addition of serum containing medium). Furthermore, for all conditions, we prepared one single “stock solution” which was used in parallel for both the luciferase assay (30 µL mix + 70 µL serum-free medium) and the clonogenic assay (300 µL mix + 700 µL serum-free medium).

**Fig. 6:**
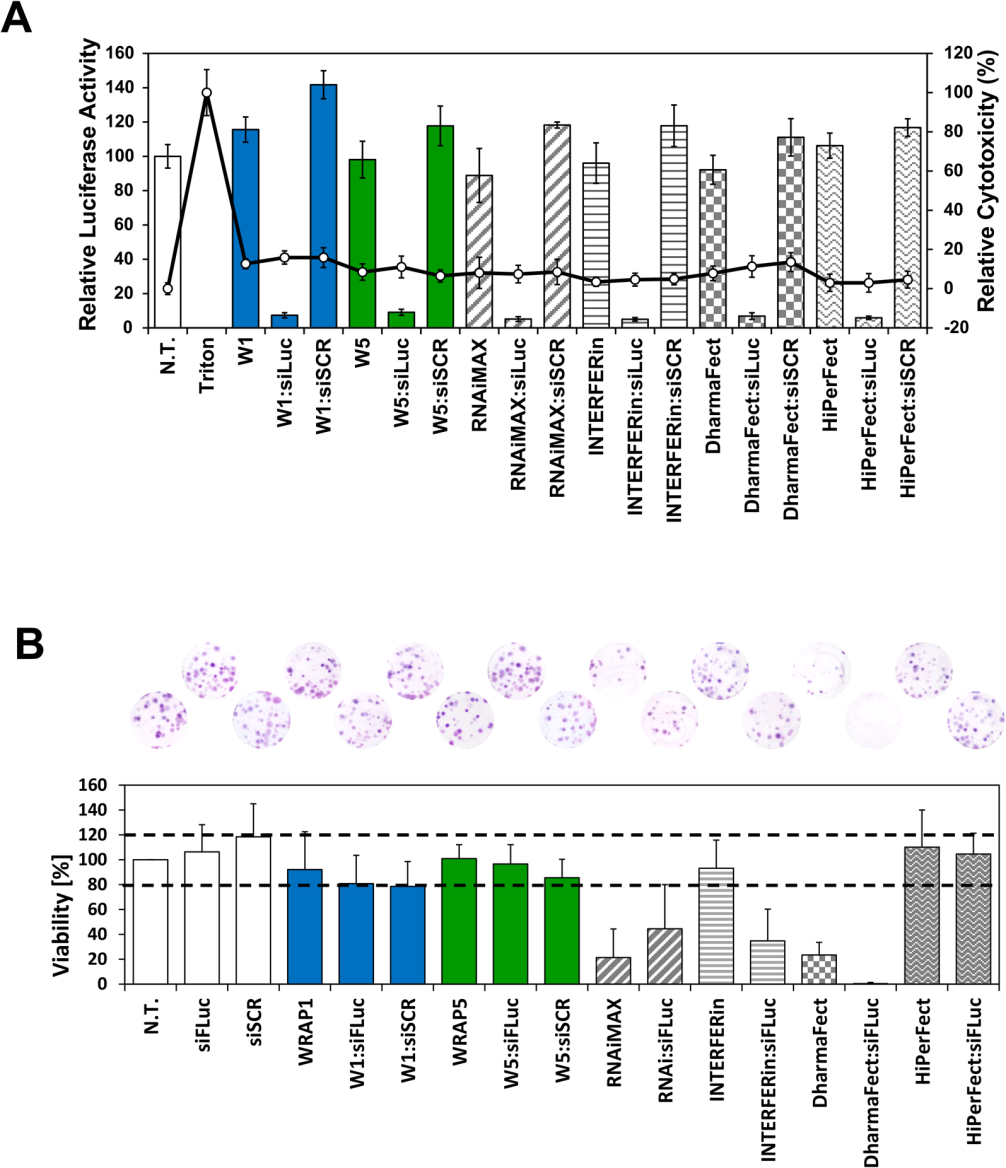
Comparison of the lead WRAP-based PBNs with other transfection reagents. **A** Relative Luc activity (%) after WRAP:siLuc PBN transfection on U87 cells compared to other lipid-based transfection reagents (siRNA = 50 nM). **B** Clonogenic assay after WRAP:siLuc PBNs transfection on U87 cells compared to other lipid-based transfection reagents (siRNA = 50 nM). Dashed lines represent the range of 100 ± 20% showing no impact on the cell viability. Abbreviations: siLuc = firefly luciferase siRNA, N.T. = non-treated cells, Ctrl = Controls. Data represent mean ± SD, with n = 2 independent experiments in triplicate for luciferase assay and n = 4 independent experiments in duplicate for clonogenic assay

Using these conditions, we first performed luciferase knock-down experiments and we observed an important cell mortality (> 80%) for most of the lipid-based conditions making impossible any comparison (Data not shown). Therefore, we slightly changed the incubation protocol by removing the whole transfection solution after 1.5 h and adding fresh serum-containing medium for 36 h, a procedure which is recommended alternatively by manufacturers for all transfection reagents (Table 1-SI). Using these conditions, cytotoxic effects of the lipid-based transfection reagents were reduced under 20% as measured by LDH assay (Fig. 6A). More importantly, we showed that all transfection reagents (peptide- or lipid-based) were identically active with a specific luciferase silencing of about > 80% using a siRNA concentration of 20 nM (Additional file 1: Figure S8). Again, no silencing was recorded with the scrambled siRNA version. In view of the clonogenic assay and in order to push the limits of the used transfection reagents, we also performed the luciferase assay using a siRNA concentration of 50 nM (Fig. 6A). We observed nearly the same specific luciferase silencing capacity as shown for the lower siRNA concentration without any cytotoxic effects for all conditions after this short-term incubation (LDH assay).

To evaluate the potential long-term cytotoxicity of the peptide- or lipid-based transfection reagents, we performed clonogenic assays over a 14 days period. This cell survival assay evaluated all modalities of cell death based on the ability of a single cell to grow as a colony over a period of two weeks. In analogy to the luciferase screening, U87 cells were incubated with the different transfection reagents using the same protocol (Fig. 6A). We determined a viability threshold corresponding to 100% ± 20% (dotted line in Fig. 6B). Under this condition, siRNA alone, W1 and W5 alone or complexed with siRNAs (siLuc or siSCR) had no impact on cell viability with a cell proliferation equal to the non-treated cells.

In contrast, we could reveal some cytotoxic effects for RNAiMAX and DharmaFect, alone or complexed with siRNA (viability ≤ 20%) under these used conditions. For INTERFERin, no cytotoxicity for the transfection reagent alone was recorded, but once complexed to siRNAs, only 30% of cell colonies survived. The only lipid-based transfection reagent without any deleterious effect on cell division was HiPerFect, whether used alone or complexed to siRNAs.

In conclusion, we evaluated 6 different peptide- or lipid-based transfection reagents for their luciferase silencing and long-term cell viability. We observed that all transfection reagents (WRAP-PBNs, RNAiMAX, INTERFERin, DharmaFect and HiPerfect) revealed the same silencing properties (> 80% using 20 nM or 50 nM siRNA) with no short-term cytotoxic effects. However, some long-term cytotoxicities were observed for RNAiMAX, INTERFERin or DharmaFect under the used conditions.

## Discussion

By performing a structure–activity relationship (SAR) study with our lead WRAP1 (W1) and WRAP5 (W5) peptides [20, 23] together with 13 newly synthesized analogues, we have evaluated the role of the leucine, arginine and tryptophan residues with the WRAP peptide sequences as well as the impact of the structural peptide changes after the addition of siRNAs.

In the first step of our SAR study, we showed the higher luciferase inhibition rate of the WRAP:siRNA PBNs compared to the C6M1 and C6M1-L peptides. Because of the close similarity in amino acid content between these two families of peptides, we wanted to understand why these peptides behaved differently in their ability to transfect siRNAs into cells. According to the publication of our colleagues [30], measurement by Dynamic Light Scattering (DLS) and microscopy techniques revealed a particle size with a diameter of 70 nm for the C6M1:siRNA in water. This diameter was closed to the diameter observed for the WRAP:siRNA PBNs (around 80 nm as shown in [20] and herein Table 1). Therefore, we cannot consider the particle size as the main criterion to explain this discrepancy in the transfection rate between these peptides. But for all our analogues evaluated in this work, it was noteworthy that PBNs with a diameter higher than about 250 nm did not induce any biological effects in our siRNA-Luciferase assay.

The clearer difference between the two series of peptides relied on the higher number of arginine in the C6M1 series (7 residues) compared to the WRAP series (4 residues). Arginine residues were expected to interact directly with the phosphate groups of siRNAs through ionic interactions during the PBNs formation. They were also responsible of the translocating process since peptides made exclusively of arginine residues (poly-Arg) were able to translocate efficiently into cells [25]. Logically, we could hypothesize the C6M1 to be more efficient regarding this aspect, but we faced exactly the opposite (Fig. 1B). One possibility remained the slower or less efficient release of the siRNA once the PBNs reached the cytosol for a peptide containing more arginine residues. This was highlighted in our experiment when heparin was used to displace the binding of siRNAs from the different peptides. The higher number of arginine residues in peptides required a higher concentration of heparin to destabilize the PBNs (Additional file 1: Figure S5). This was coherent with results published by Jafari et al*.* for C6M1 (8 positive charges) (see Fig. 7 of the publication [30]) showing ~ 20% siRNA recovery after heparin addition (5 µg) in a gel-shift retardation assay corresponding to our heparin incubation (1 eq. = 5.5 µg) with WRAP1 (5 positive charges) having a siRNA recovery of ~ 50%. siRNA recovery from peptides with higher positive charges needed higher amount of heparin.

In line with this, Jafari and colleagues [21] also demonstrated that the molar ratio of C6M1 peptide required to completely encapsulate siRNAs was in the same range than the molar ratio used with our WRAP peptides (both requiring an optimal ratio of peptide over the siRNA of R = 15 as observed in a gel-shift retardation assay in our respective laboratories). In our comparative experiment, we used the same ratio for both peptide series. Consequently, the charge ratio between C6M1 and WRAP peptides over the siRNAs can be calculated as being 3.5 cations/anion for C6M1 and 2.2 cations/anion for WRAP because of the presence of 7 arginine residues in C6M1 versus 5 in the WRAP peptides. There was thus an excess of arginine residues over anionic groups from the siRNA in the C6M1 complexes thus possibly impairing an efficient cellular release of the siRNAs from the PBNs. We cannot exclude this difference in the lower inhibitory response of C6M1 to be the direct consequence of its higher arginine content.

Moreover, we also observed that the deletion of one single leucine residue from a leucine doublet within the C6M1 peptide (C6M1-L) could reduce of about 50% the luciferase silencing mediated by the parental peptide. Therefore, we also wanted to verify whether the leucine doublets, mainly present in our WRAP peptides at both extremities, but also in the middle of the sequence, were important in the PBN formation as well for the siRNA-induced luciferase silencing. Despite the ability for all these ∆-Leu peptides to bind siRNAs with roughly the same level in a gel-shift assay (Fig. 2A), their respective ability to induce a biological silencing effect was rather different (Fig. 4A). W1 peptide deleted from the doublets at the extremities (W1-2L) was still active, while the deletion of leucine residues doublets at any position in W5 was highly deleterious (Fig. 4A). In these latter cases however, the size of the formed nanoparticles with the W5 peptide series led to aggregates with a diameter higher than 1,000 nm, while the PBNs made with the W1 peptide deleted from its leucine (W1-2L and W1-Lm) remained in the 100 nm range. Despite the ability of these peptides to also induce the expected biological response, we noticed their instability upon 4 days storage at 4 °C. Leucine doublets thus appeared to positively influence the formation of PBNs with a size acceptable for promoting the intracellular transport of siRNAs.

We also related the efficacy of our WRAP peptides and analogues with the length of the helical structure formed once the peptides were combined with the siRNAs. The length of the helix was also directly correlated with the size of the resulting PBNs showing that peptides structured in short helix (below 2 helical turns) formed bigger PBNs. The single exception was the peptide W1-1P (with a proline residue in the middle of the sequence) having a short helix (only 1.4 helical turn) and forming 150 nm sized PBNs. However, this PBN has a weak ability to extinguish the targeted luciferase expression in our transfection assay (only 37% with 10 nM siLuc) (Fig. 5B). Additionally, compared to the other peptides, that one was strongly unstable upon a 4 day-period (Table 1), while parental W1 and W5 peptides were stable at least for weeks (Additional file 1: Figure S1). Finally, we believe that the defined balance between hydrophobicity and charges of the peptide sequence will have a higher impact on transfection activity than helical structuration.

In our previous work [20], we showed that the number of tryptophan residue was important to promote PBN formation and subsequently, the extinction of the targeted protein expression. Herein, we investigated whether their location within the sequence could influence the overall efficacy of the transfection. With the exception of W5-mix (not stable and showing low silencing), we did not observe a spectacular effect associated with the relative tryptophan position within the WRAP sequences.

Based on the results described in this work and those obtained previously [20], we established for WRAP peptides and WRAP-PBNs the following consensus motif LL-[X]n-LL with n = 11–12 amino acids including the three restrictions for X:

- The number of internal arginine residues should be n = 4
- The number of internal leucine residues should be n = 4 and they required to be inserted as doublet.
- The number of internal tryptophan residues should be n = 3 for a grouped tryptophan localization in the middle of peptide sequence (WWW) or n = 4 for a grouped (2 × WW) or a clustered through secondary structure folding (4 × single W).

Further rules concerning the amino acid composition are difficult to postulate. To obtain stable PBNs with a maximal silencing activity (~ 80% or more), we recommend to use PBNs with a mean size between 80 and 110 nm as well as a minimal helix turn of 2.5 because the helix length seems to positively influence the PBN mean size (e.g. W1-4R: 1.9 helix turn, 441 nm versus W5-6R: 3.1 helix turn, 64 nm). However, the helix length did not automatically influence the PBN stability as shown for W1-2L which is not stable after a 4 day-storage at 4 °C.

Finally, because FDA approved nucleic acid therapeutics (siRNA and mRNA) are delivered by lipid-based nanoparticles [35], we compared our WRAP-PBNs to four commercially available transfection reagents. If the overall efficacy of the WRAP peptides and the non-peptide transfection reagents could be comparable over a short-term period, we noticed some differences in cell viability in a long-term clonogenic assay. Only one out of the four reagents we tested showed none toxicity as recorded for the WRAP peptides. Therefore, researcher should be aware of the interpretation of their protein silencing due to potent long-term cytotoxic effect or other side effects such as alterations of the lipidome of hepatocytes [36] and of Hela cells [37] or markedly inhibit cholesterol biosynthesis [38] by some of the lipid-based transfection reagents.

In conclusion, WRAP peptides could be used as efficient and safe compounds to extinguish the expression of any kind of endogenous or exogeneous proteins with a wide number of cell-types [20] as well as in vivo in solid tumors as recently demonstrated by our group [39].

## Materials and methods

### Materials

WRAP peptides were synthesized on the SynBio3 platform (IBMM Montpellier) and crude products were purified in house following a qualitative analysis by HPLC/MS. siRNA (siFLuc: 5’-CUU-ACG-CUG-AGU-ACU-UCG-AdTdT-3’ as sense strand and siSCR: 5’-GAA-UGC-GAC-UCA-UGA-AGC-UdTdT-3’) were purchased from Eurogentec. The following siRNA transfection reagents were used: RNAiMax (Life Technologies), INTERFERin (PolyPlus), DharmaFECT (Dharmacon) and HiPerFect (Qiagen).

### Nanoparticle formation

Stock solutions of WRAP peptides (see Table 1) and of siRNA were prepared in pure water and in RNase-free water, respectively. Nanoparticles were formulated in pure water supplemented by 5% (m/v) glucose by adding first the CPP and then the corresponding amount of siRNA at molar ratio (R) of 20 (WRAP:siRNA = 20:1) at room temperature. Formulated PBNs could be stored for several weeks at 4 °C without loss of transfection efficacy.

### Peptide structure prediction

PEPstrMOD server was used to predict the secondary structure of WRAPs (http://osddlinux.osdd.net/raghava/pepstrmod/) [31, 32].

### Circular dichroism (CD) measurements

CD spectra were recorded on a Jasco 810 (Japan) dichrograph in quartz suprasil cells (Hellma) with an optical path of 1 mm for peptide in solution or in the presence of liposomes vesicles. Same peptide concentrations (10 µM) were used for each condition. Spectra were obtained from 3 accumulations between 190 and 260 nm with a data pitch of 0.5 nm, a bandwidth of 1 nm and a standard sensitivity.

### Agarose gel-shift retardation assay

WRAP:siRNA complexes [siRNA = 3.5 µM in 20 µL of an aqueous solution containing 5% glucose] were formed at different ratios and pre-incubated for 30 min at room temperature. Then 20 µL were loaded on an agarose gel (1% w/v, Sigma-Aldrich) and electrophoresis was performed at 50 V for 25 min. To visualize the siRNA the agarose gel was stained with GelRed (Interchim) for UV detection and images were acquired using the Amersham 600 imager (GE Lifesciences). The signal intensities were quantified after background subtraction using Image J software (gel analyze tool). Each band intensity corresponding to a distinguished condition was then normalized to the band intensity of the siRNA alone (= 100%): Relative fluorescence (%) = fluorescence intensity (condition x)/fluorescence intensity (siRNA alone) × 100.

### Dynamic light scattering (DLS)

WRAP:siRNA nanoparticles (WRAP = 10 µM, siRNA = 500 nM, R = 20) were evaluated with a Zetasizer NanoZS (Malvern) in terms of mean size (Z-average) of the particle distribution and of homogeneity (PDI). All results were obtained from three independent measurements (three runs for each measurement at 25 °C).

### Cell culture conditions

Human glioblastoma cell line (U87) overexpressing the firefly and nanoluc luciferase (FLuc-NLuc) were kindly provided by Dr. Franck Couillaud (Bordeaux, France). Cells were grown in DMEM-pyruvate-GlutaMAX™ medium (Life Technologies), supplemented with 1% penicillin/streptomycin (Life Technologies), 10% heat-inactivated fetal bovine serum (FBS, ThermoFisher), 0.1% non-essential amino acids (NEAA 1X, LifeTechnologies) and 0.01% hygromycin B (50 µg/mL, Invitrogen). Cells were maintained in a humidified incubator with 5% CO_2_ at 37 °C.

### Luciferase assay

U87 cells (5000 cells/well) were seeded 24 h before experiment into 96-well plates. Before PBN incubation, the cell growth medium was replaced by 70 µL of fresh pre-warmed serum-free DMEM. Afterwards, 30 µL of the PBN solutions were added directly to the medium recovering the cells and incubated 1.5 h at 37 °C. Finally, 100 µL fresh DMEM supplemented with 20% FBS were added to the cells (10% FBS at final concentration) and the cells were incubated for further 36 h. The medium covering the cells was then carefully removed and 50 µL of 0.5 X Passive Lysis Buffer (PLB; Promega) were added for 30 min cell lysis at 4 °C. After a centrifugation step (10 min, 1,800 rpm, 4 °C), 10 µL of each cell lysate supernatant were transferred into a white 96-well plate (Greiner Lumitrac™ 200). Luciferase activity was quantified using a plate-reading luminometer (POLARstar Omega, BMG Labtech) and half-diluted dual luciferase assay reagents as described by the manufacturer (Promega). The results were expressed as percentage of relative light units (RLU) for each luciferase, normalized first to non-treated cells (%FLuc and %NLuc) and then to the value of %NLuc to obtain the relative Luc activity (%FLuc/%NLuc).

### Cytotoxicity assay

The Cytotoxicity Detection Kit^Plus^ (LDH, Roche Diagnostics) was used to evaluate the cytotoxicity induced by the PBNs. After the PBN incubation (36 h), at least one well was used as a LDH positive control (100% toxicity) by adding Triton X-100 (Sigma-Aldrich) to a final concentration of 0.1% (~ 15 min incubation at 37 °C). Afterwards, 50 µL supernatant of each well were transferred in a new clear 96-well plate (Greiner) and completed with 50 µL/well of the “dye solution/catalyst” mixture as recommended by the manufacturer. The plate was then incubated in the darkness for 30 min at room temperature. Reaction was stopped by adding 25 µL/well of HCl (1 N) before measuring the absorption at 490 nm using the POLARstar Omega plate reader (BMG Labtech). Relative toxicity (%) was calculated with the following formula: [(exp. value–value non-treated cells)/(value triton–value non-treated cells)] × 100.

### Comparison with other transfection reagents

#### Complex formation

For a transfection mix of 300 µL, 5 µL siRNA [10 µM] were mixed with 4.4 µL WRAP [200 µM], 7.5 µL RNAiMax, 12 µL INTERFERin, 10 µL DharmaFect, 9 µl for HiPerFect. These transfection mixes were diluted in serum-free medium (1:3.33) in order to obtain a final siRNA concentration of 50 nM.

#### Luciferase assay

U87 cells (5,000 cells per well) were seeded 24 h before experiment into 96-well plates. Before PBN or lipid-based transfection reagent incubation, the cells were washed twice with D-PBS (Life Technologies). 70 µL of fresh pre-warmed serum-free DMEM and 30 µL transfection solutions were added to the cells and incubated 1.5 h at 37 °C. Afterwards, solutions recovering the cells were replaced by 200 µL fresh DMEM supplemented with 10% FBS and the cells were incubated for further 36 h at 37 °C. At the end of the incubation, luciferase activity was measured as described above.

#### Clonogenic assay

U87 cells (450 cells per well) were seeded 24 h before experiment into 6-well plates. Before PBN or lipid-based transfection reagent incubation, the cells were washed twice with D-PBS (Life Technologies). 700 µL of fresh pre-warmed serum-free DMEM and 300 µL transfection solutions were added to the cells and incubated 1.5 h at 37 °C. Afterwards, solutions recovering the cells were replaced by 2000 µL fresh DMEM supplemented with 10% FBS and the cells were incubated for further 10 days at 37 °C. At the end of the incubation, cells were first fixed using a solution of methanol/acetic acid (3:1) at 4 °C for 20 min. The fixed cells were colored using a freshly prepared Giemsa solution (Sigma-Aldrich) diluted in water (3.5:10) for 20 min at room temperature. To visualize the colonies, each well was abundantly washed with water and then dried. Images of the colonies were acquired using the Amersham 600 imager (GE Lifesciences). Colonies for each well were counted and normalized to the non-treated cells.

## Supplementary Information

**Additional file 1: Table S1.** Conditions for siRNA transfection from commercially available transfection reagents. **Fig. S1.** Evaluation of the long-term stability of the WRAP-PBNs by measuring their luciferase activity. **Fig. S2.** CD spectra of WRAP1 and its analogues as described in Table 1. **Fig. S3.** CD spectra of WRAP5 and its analogues as described in Table 1. **Fig. S4.** Comparison of luciferase silencing of WRAP-based NPs depending on their formulation conditions. **Fig. S5**. Effect of heparin on the stability of siRNA-loaded nanoparticles by gel shift assay. **Fig. S6.** Evaluation of the cellular siRNA internalization by WRAP peptides and their analogues. **Fig. S7.** Evaluation of the WRAP peptides and their analogues in terms of cell cytotoxicity and viability. **Fig. S8.** Comparison of the lead WRAP-based PBNs with other transfection reagents.
