# Supplementary material for: WRAP-based nanoparticles for siRNA delivery: a SAR study and a comparison with lipid-based transfection reagents
Source: J Nanobiotechnology. 2021 Aug 11;19:236. doi: 10.1186/s12951-021-00972-8 (PMC8359084; doi:10.1186/s12951-021-00972-8)
Supplement: Supplementary file 1 — Additional file 1: Table S1. Conditions for siRNA transfection from commercially available transfection reagents. Fig. S1. Evaluation of the long-term stability of the WRAP-PBNs by measuring their luciferase activity. Fig. S2. CD spectra of WRAP1 and its analogues as described in Table 1. Fig. S3. CD spectra of WRAP5 and its analogues as described in Table 1. Fig. S4. Comparison of luciferase silencing of WRAP-based NPs depending on their formulation conditions. Fig. S5. Effect of heparin on the stability of siRNA-loaded nanoparticles by gel shift assay. Fig. S6. Evaluation of the cellular siRNA internalization by WRAP peptides and their analogues. Fig. S7. Evaluation of the WRAP peptides and their analogues in terms of cell cytotoxicity and viability. Fig. S8. Comparison of the lead WRAP-based PBNs with other transfection reagents. [file 12951_2021_972_MOESM1_ESM.docx]

**WRAP-based nanoparticles for siRNA delivery: A SAR study and a comparison with lipid-based transfection reagents.**

Karidia Konate^1^, Emilie Josse^1^, Milana Tasic^1^, Karima Redjatti^1^, Gudrun Aldrian^2^, Sébastien Deshayes^1^, Prisca Boisguérin^1^, Eric Vivès^1*^.

^1^ PhyMedExp - Université de Montpellier, INSERM U1046, CNRS UMR 9214, CHU Arnaud de Villeneuve, 371 av. doyen Giraud, 34295 Montpellier Cedex 5, France

^2^ Sys2Diag, UMR 9005-CNRS/ALCEDIAG, 1682 Rue de la Valsière, 34184 Montpellier CEDEX 4, France

* Corresponding author: Eric Vivès, phone: 0033 4 67 415224. Email: eric.vives@umontpellier.fr

**MATERIALS AND METHODS**

***Confocal microscopy:*** U87 cells (150,000 cells/well) were seeded 24 h before experiment into 12-well plates having glass coverslips in the bottom of each well. Before PBN incubation, the cell growth medium was replaced by 350 µL of fresh pre-warmed serum-free FluoroBrite DMEM. Afterwards, 150 µL of the PBN solutions [CPP = 400 nM, siRNA-Alexa488 = 20 nM] were added directly to the medium recovering the cells and incubated 1.5 h at 37°C.

5 min before the end of the PBN incubation, 1 μg/mL Hoechst 33342 (Sigma-Aldrich) and 1x CellMask™ Deep Red for nuclei and cell labeling, respectively, were added in each well. After removal of the stained solution, the coverslips were rinsed three times with PBS. Cells were fixed during 10 minutes with a 2% Paraformaldehyde solution, rinsed two times with PBS, then coverslips were mounted immediately.

Images were acquired on a Zeiss LSM 800 confocal microscope (Zeiss Apo 63×/1.2 W DICIII objective lens; Hoechst 33342: λex=405 nm/λem=400–497 nm; Dep Red: λex=640 nm/λem=650-700 nm; Alexa488: λex=488 nm/λem=400-539 nm). Image acquisition was done sequentially to minimize crosstalk between the fluorophores. Same laser power and master gain were used to record all images. Each confocal image was merged and adjusted with the same brightness and contrast parameters using the Fiji software.

***Crystal violet assay:*** U87 cells (20,000 cells/well) were seeded 24 h before experiment into 96-well plates. Before PBN incubation, the cell growth medium was replaced by 70 µL of fresh pre-warmed serum-free DMEM. Afterwards, 30 µL of the peptide solutions were added directly to the medium recovering the cells and incubated 1.5 h at 37°C (final concentrations of 2 µM, 1 µM, 0.5 µM and 0.125 µM) Finally, 100 µL fresh DMEM supplemented with 20% FBS were added to the cells (10% FBS at final concentration) and the cells were incubated for further 24 h.

The next day, add in the corresponding wells 20 µL of 1x Triton-X100 solution as negative control. After 15 min of Triton-X100 incubation, cells were washed twice with 100 µL PBS and fixed with 100 µL 4% paraformaldehyde solution for 10 min. Then, cells were rinsed twice with 100 µL PBS and stained with 100 µL 0.2% crystal violet solubilized in 10% ethanol for 10 min. Staining was removed by washing the cells 5x with 100 µL PBS before let them drying. To quantify crystal violet, cells were solubilized with an Infinite M200 pro aqueous solution of 10% acetic acid (200 µL) followed by the absorbance measurement at 590 nm using a multiplate reader (Tecan). Viability was calculated using the following equation:

% viability = ((Abs Sample – Abs Triton) / (Abs NT – Abs Triton)) * 100

**Table S1: Conditions for siRNA transfection from commercially available transfection reagents.**

*Footnotes: / = no information available.*


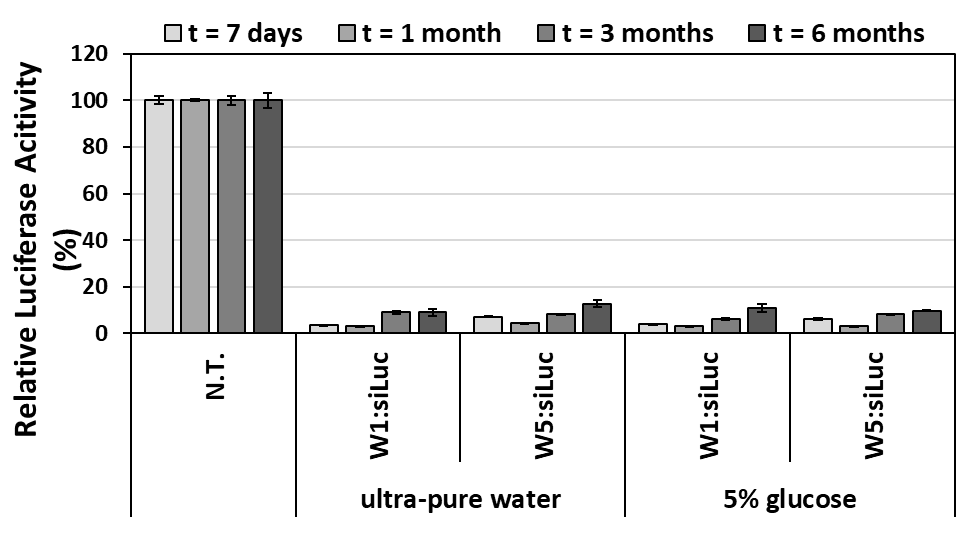


**Figure S1: Evaluation of the long-term stability of the WRAP-PBNs by measuring their luciferase activity.**

Graphical representation highlighted the relative Luc activity (%) after transfection with WRAP:siLuc complexes on U87 cells. Conditions: WRAP:siRNA (R = 20) with [WRAP] = 400 nM and [siRNA] = 20 nM. Abbreviations: siLuc= firefly luciferase siRNA, N.T. = non-treated cells, Ctrl = Controls. Data represent mean ± SD, with n = 2 independent experiments in triplicate.


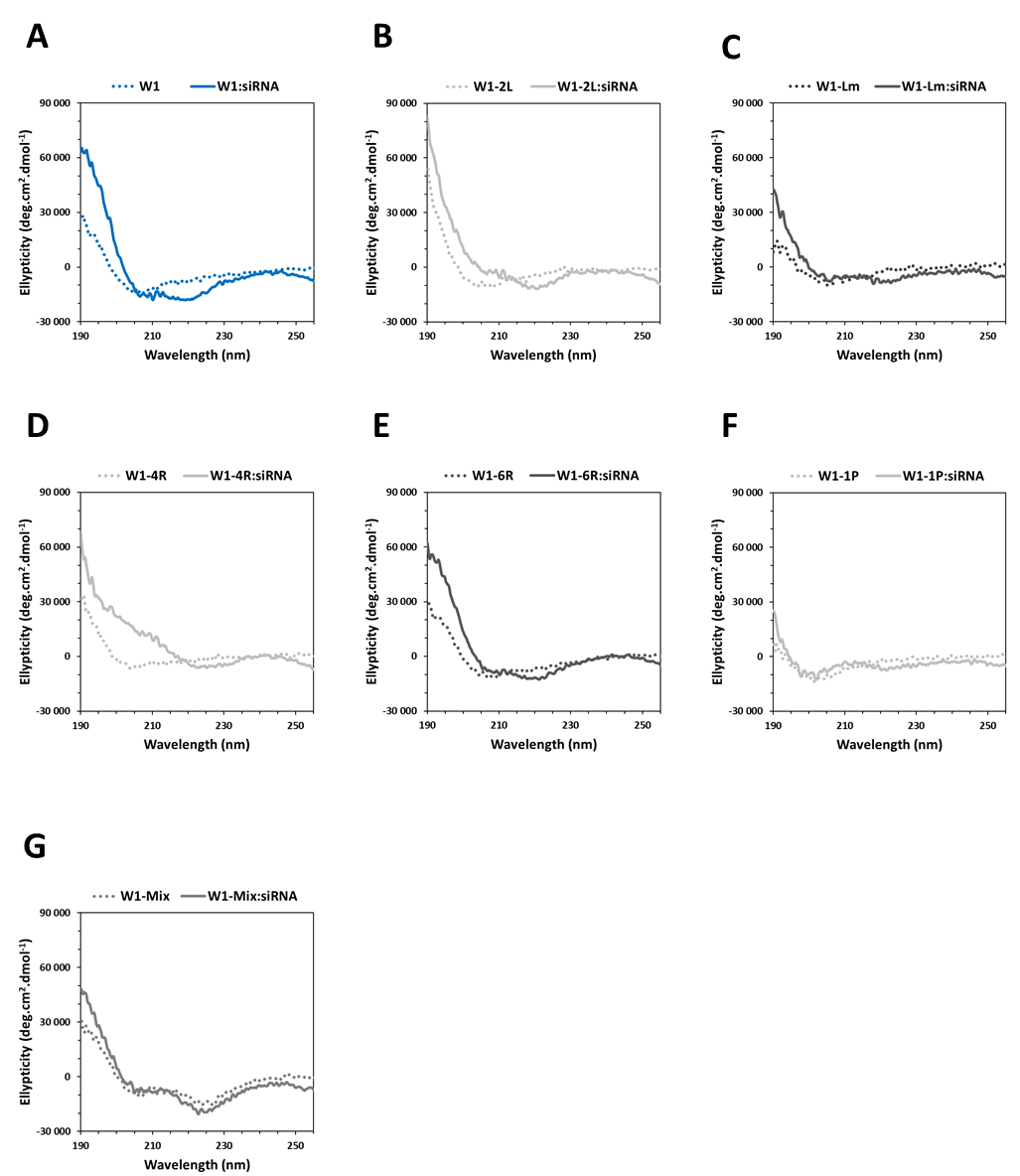


**Figure S2: CD spectra of WRAP1 and its analogues as described in Table 1.**

Experimental conditions: CPP alone (10 µM), CPP:siRNA complexes (R = 20, [CPP] = 10 µM, [siRNA] = 0.5 µM).


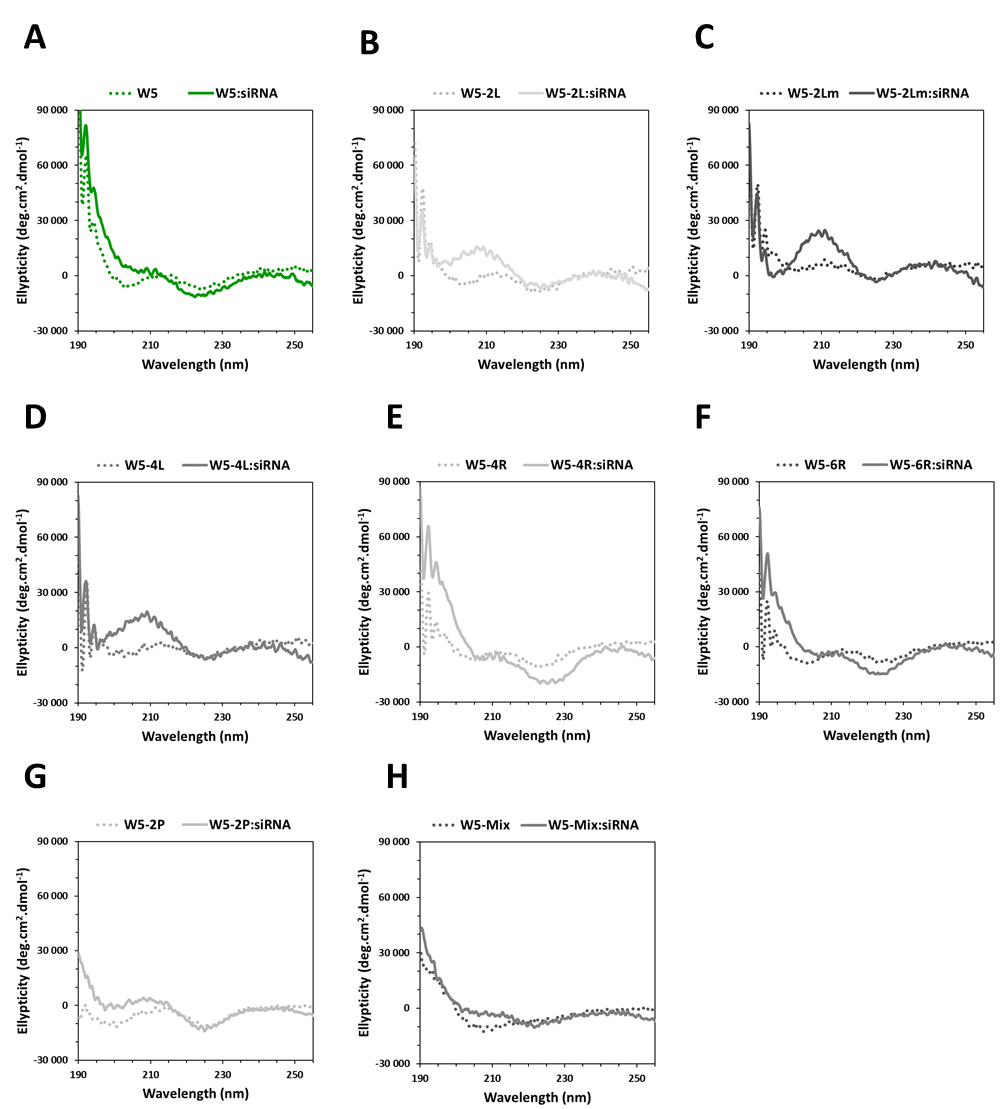


**Figure S3: CD spectra of WRAP5 and its analogues as described in Table 1.**

Experimental conditions: CPP alone (10 µM), CPP:siRNA complexes (R = 20, [CPP] = 10 µM, [siRNA] = 0.5 µM).


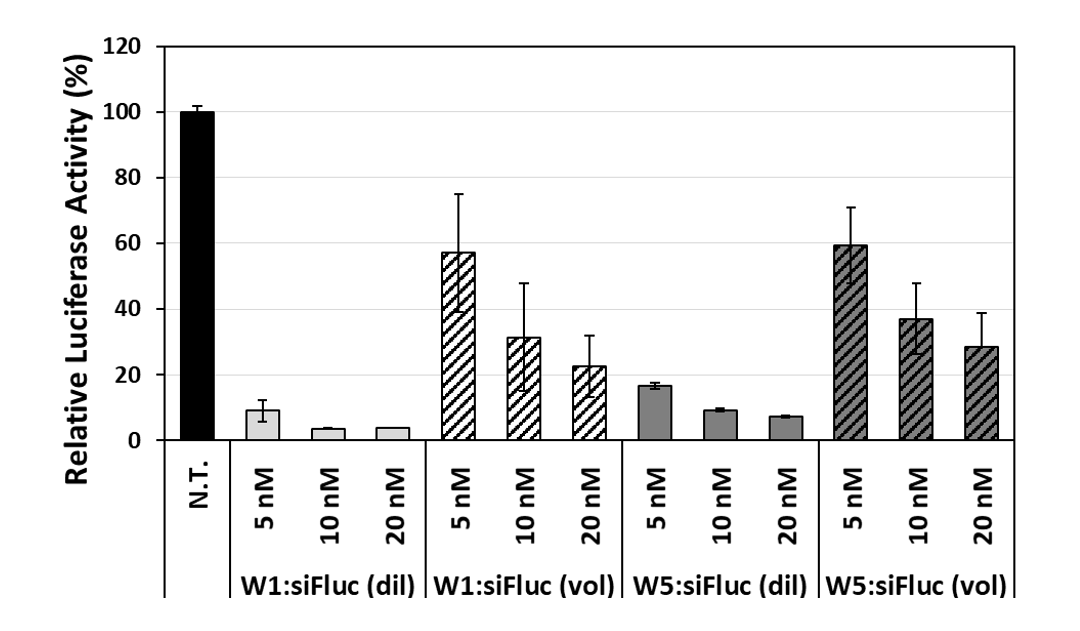


**Figure S4: Comparison of luciferase silencing of WRAP-based NPs depending on their formulation conditions.**

WRAP:siRNA PBNs were formulated using a “stock” solution which is diluted at the required concentration (= (dil) in the graph) or using a WRAP:siRNA “volume-to-volume” dilution at the required concentration (= (vol) in the graph).

Graphical representation highlighted the relative Luc activity (%) and relative cytotoxicity (LDH quantiﬁcation, %) after transfection with WRAP:siFLuc complexes on U87 cells. Conditions: WRAP:siRNA (R = 20) at the indicated siRNA concentrations. Abbreviations: siFLuc= firefly luciferase siRNA, N.T. = non-treated cells. Data represent mean ± SD, with n = 2 independent experiments in triplicate.


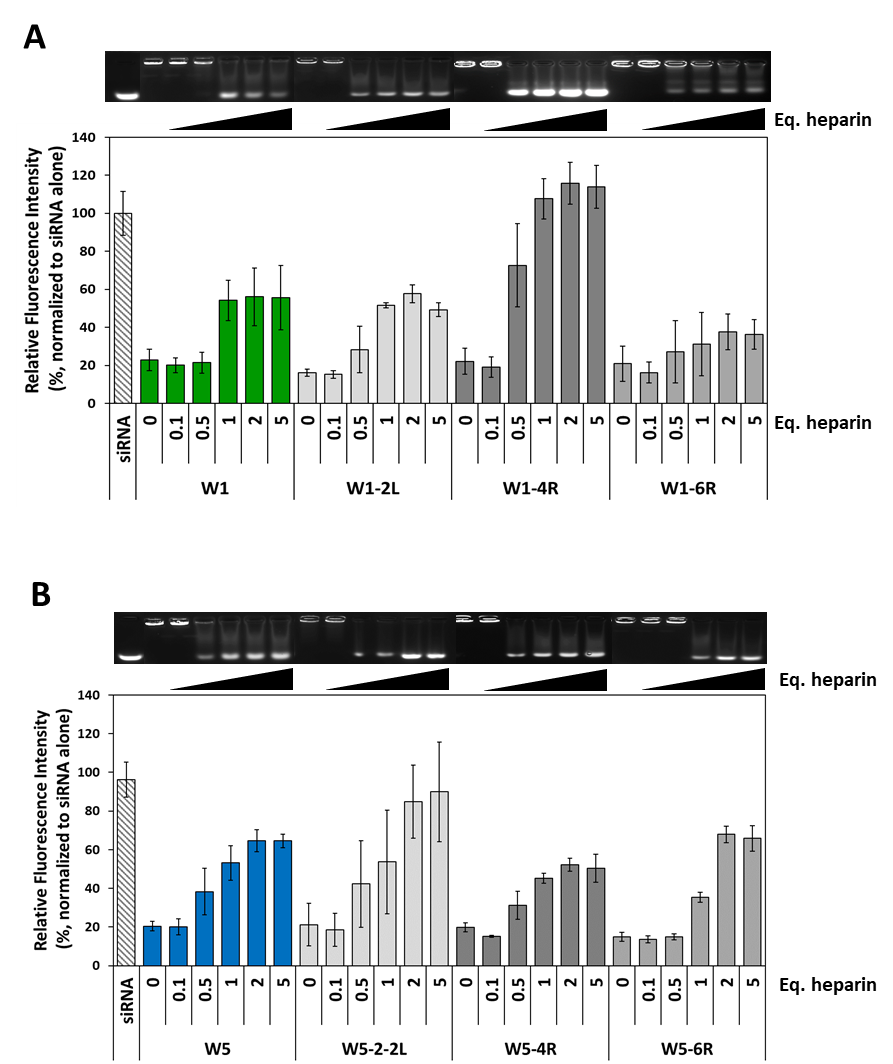


**Figure S5: Effect of heparin on the stability of siRNA-loaded nanoparticles by gel shift assay.**

Pre-formed WRAP:siRNA complexes at R20 (CPP = 100 µM, siRNA = 5 µM) were incubated with heparin at different molar equivalents (0.1, 0.5, 1, 2 and 5 eq.) of siRNA concentration and thereafter analyzed by electrophoresis on agarose gel (1 % wt/vol) stained with GelRed. Data represent: mean ± SD, with n = 3.


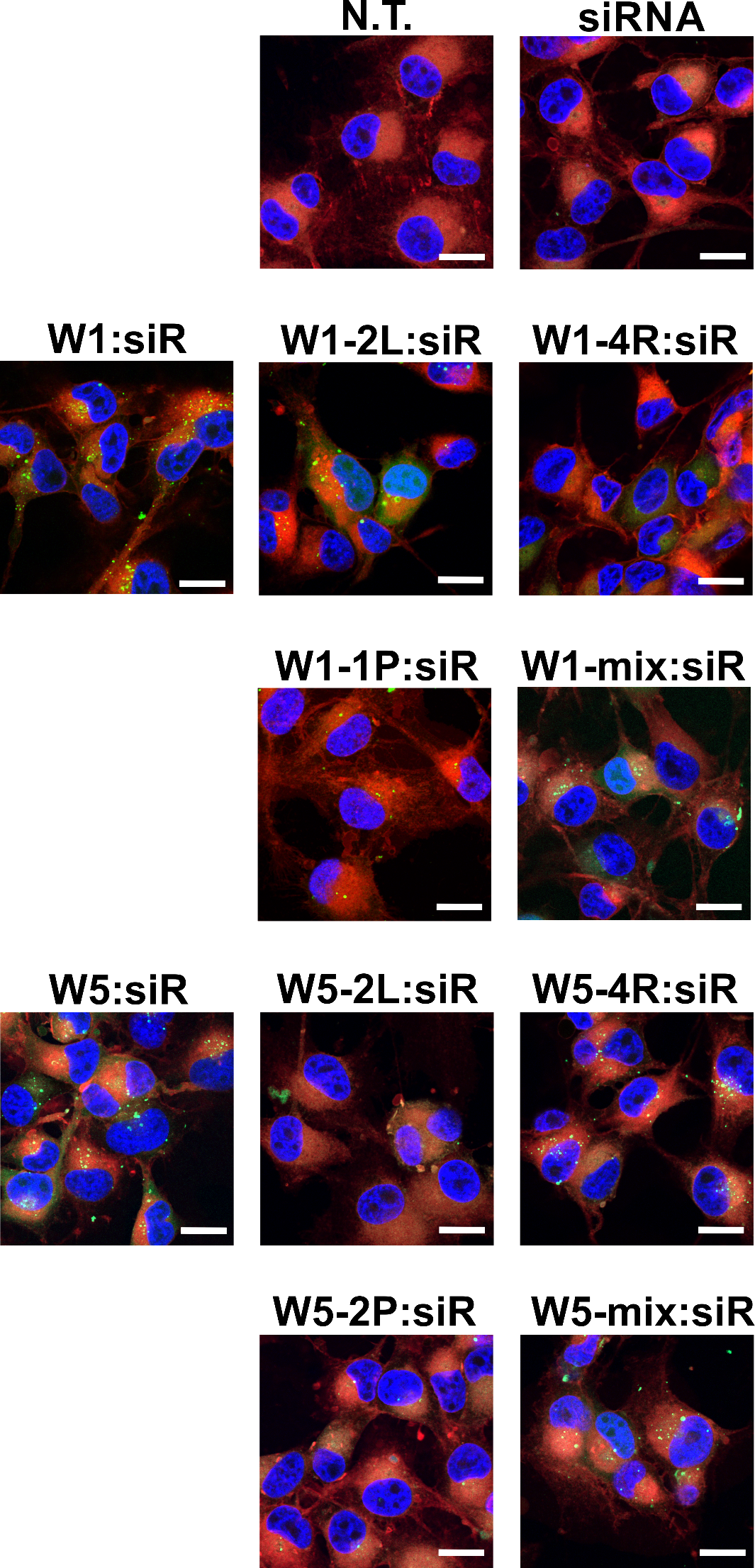


**Figure S6: Evaluation of the cellular siRNA internalization by WRAP peptides and their analogues.**

Fixed U87 cells were visualized by confocal microscopy after 1.5 h incubation with 5% glucose (non-treated cells, N.T.), with siRNA-Alexa488 alone (negative control, 20 nM) and with the indicated WRAP:siRNA (CPP = 400 nM, siRNA--Alexa488 = 20 nM, green) in FluoroBrite DMEM, Hoechst dye (blue) and CellMask Dep Red (red). White bars represent 20 μm.


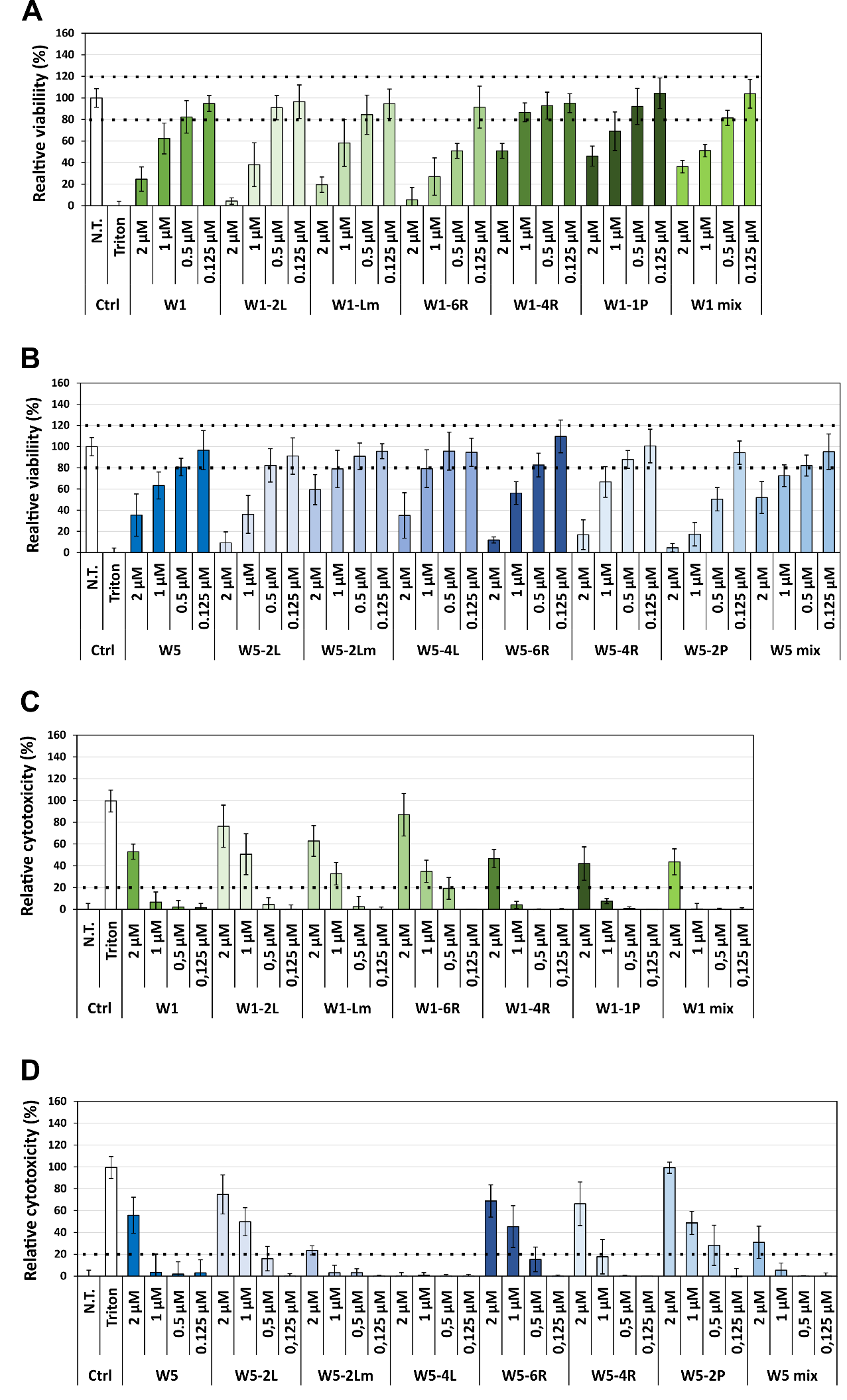


**Figure S7: Evaluation of the WRAP peptides and their analogues in terms of cell cytotoxicity and viability.**

(**A, B**) Relative viability (%) after WRAP analogues transfections on U87 cells compared to the parental WRAP peptide at the indicated concentrations. Cell viability was determined by crystal violet assays on three independent experiments performed in triplicates. Dotted lines (100% ± 20%) represented viable cells.

(**C, D**) Relative cytotoxicity (%) after WRAP analogues transfections on U87 cells compared to the parental WRAP peptide at the indicated concentrations. Cell cytotoxicity was determined by LDH assays on three independent experiments performed in triplicates. Below dotted line (20%), no cytotoxicity is observed.

A concentration between 2 µM and 0.125 µM was used which correspond to peptide concentrations used to formulate siRNA at concentrations between 100 nM and 6 nM.


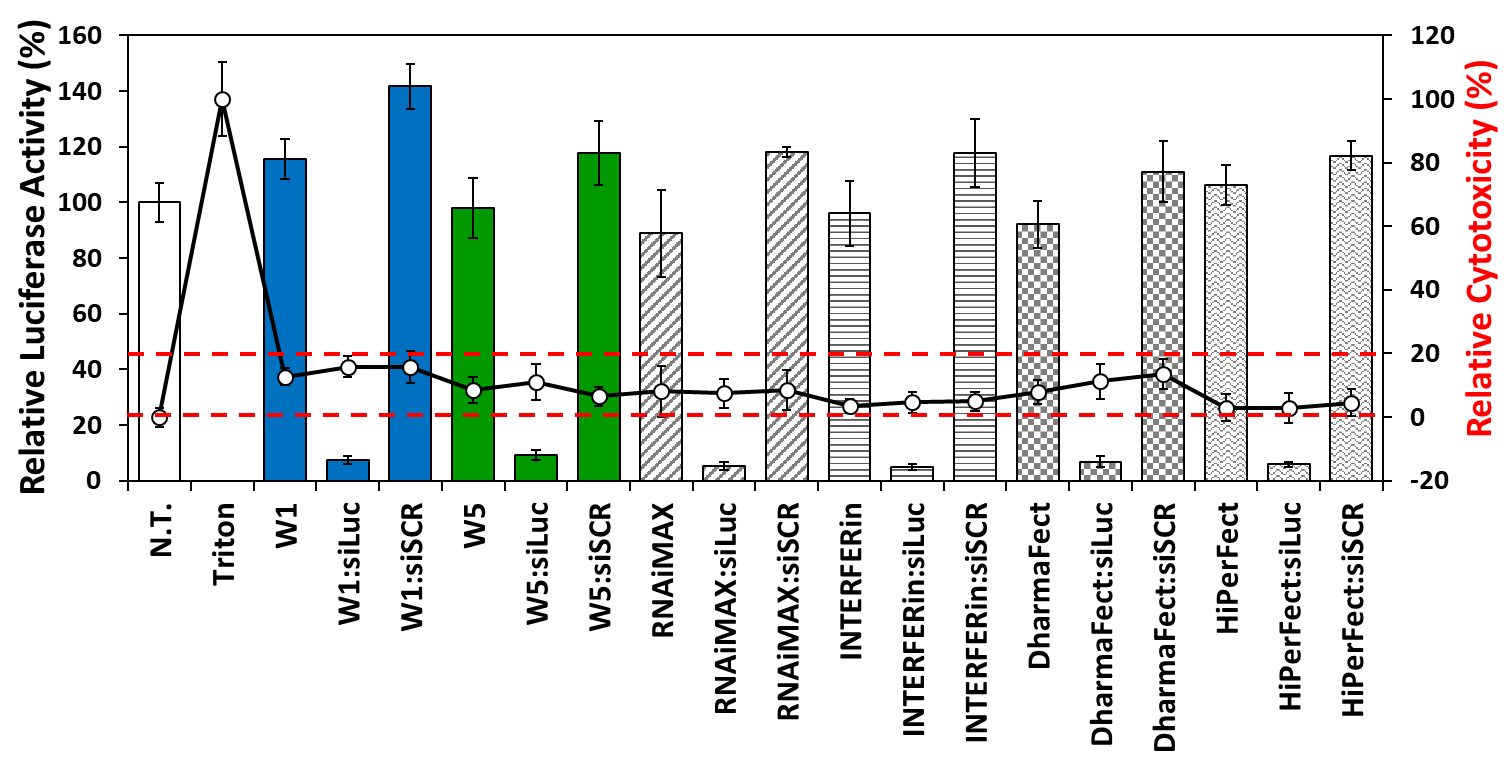


**Figure S8: Comparison of the lead WRAP-based PBNs with other transfection reagents.**

Relative Luc activity (%) after WRAP:siLuc PBN transfection on U87 cells compared to other lipid-based transfection reagents (siRNA = 20 nM).
